# A systematic review of the health and well-being impacts of school gardening: synthesis of quantitative and qualitative evidence

**DOI:** 10.1186/s12889-016-2941-0

**Published:** 2016-03-25

**Authors:** Heather Ohly, Sarah Gentry, Rachel Wigglesworth, Alison Bethel, Rebecca Lovell, Ruth Garside

**Affiliations:** European Centre for Environment and Human Health, University of Exeter Medical School, Truro, Cornwall UK; Norfolk and Norwich University Hospitals NHS Foundation Trust, Norwich, Norfolk UK; NIHR CLAHRC South West Peninsula (PenCLAHRC), University of Exeter Medical School, Exeter, Devon UK

**Keywords:** School, Gardens, Systematic review, Health, Well-being, Mixed methods

## Abstract

**Background:**

School gardening programmes are increasingly popular, with suggested benefits including healthier eating and increased physical activity. Our objectives were to understand the health and well-being impacts of school gardens and the factors that help or hinder their success.

**Methods:**

We conducted a systematic review of quantitative and qualitative evidence (PROSPERO CRD42014007181). We searched multiple databases and used a range of supplementary approaches. Studies about school gardens were included if they reported on physical or mental health or well-being. Quantitative studies had to include a comparison group. Studies were quality appraised using appropriate tools. Findings were narratively synthesised and the qualitative evidence used to produce a conceptual framework to illustrate how benefits might be accrued.

**Results:**

Evidence from 40 articles (21 quantitative studies; 16 qualitative studies; 3 mixed methods studies) was included. Generally the quantitative research was poor. Evidence for changes in fruit and vegetable intake was limited and based on self-report. The qualitative research was better quality and ascribed a range of health and well-being impacts to school gardens, with some idealistic expectations for their impact in the long term. Groups of pupils who do not excel in classroom activities were thought to particularly benefit. Lack of funding and over reliance on volunteers were thought to threaten success, while involvement with local communities and integration of gardening activities into the school curriculum were thought to support success.

**Conclusion:**

More robust quantitative research is needed to convincingly support the qualitative evidence suggesting wide ranging benefits from school gardens.

## Background

School gardening and food growing have become popular activities in thousands of schools around the world. National school gardening programmes exist in some countries, such as the Royal Horticultural Society Campaign for School Gardening in the UK [1] and the Stephanie Alexander Kitchen Garden Program in Australia [2]. The individuals and organisations behind these programmes believe that school gardening has the potential to improve children’s health, social development and academic attainment.

Since the 1990s, an increasing number of research studies have attempted to evaluate the effectiveness of school gardening programmes. More recently, several reviews of the literature on school gardening have been published [3–8]. Five of these reviews were limited to US studies and, whilst they found some empirical evidence for the health and well-being impacts of school gardening, some of their conclusions were based on theoretical rationale [3–7]. They recommended further research, using more rigorous study designs, on the effectiveness of school gardening programmes, mediation pathways and implementation factors.

The most comprehensive review, by the National Foundation for Education Research, included international studies and found evidence for positive impacts of growing activities on pupils’ nutrition and attitudes towards healthy eating [8]. It also concluded there was modest evidence for social well-being benefits, especially for lower ability pupils or those who have become disengaged from learning. This review was described as being “underpinned by a systematic process for searching, selection, screening, coding, appraisal and synthesis” (page 3). However, it did not clearly and consistently report methods (inclusion criteria; quality appraisal criteria) and results (CONSORT flow diagram; number of studies with health and well-being outcomes; description of included studies – design, methods, quality etc.) as would be expected in a systematic review. In addition, since 2011, more studies have been published on the impact and meaning of school gardening programmes, including a large cluster randomised controlled trial of the Royal Horticultural Society Campaign for School Gardening in the UK [9, 10] and several qualitative studies from the UK and the US [11–13]. Therefore, it was justified and timely to conduct a robust, mixed methods systematic review of the health and well-being impacts of school gardening, to support and inform the further development of this popular school-based intervention. This review aims to answer the following questions:

- What are the health and well-being impacts of school gardens?
- Are there different impacts for different age groups?
- What are the effects on other family and community members?
- What do school gardens mean to those who use them?
- Are there any factors that help or hinder the successful development, use or sustainability of school gardens?

## Methods

We conducted a systematic review of quantitative and qualitative literature according to a pre-specified protocol that was registered with the International Prospective Register of Systematic Reviews (PROSPERO: CRD42014007181). We used the methods of thematic synthesis described by J. Thomas and A. Harden [14]. As this was an evidence synthesis of existing research, ethical approval was not required.

### Search strategy

A search strategy was devised by the research team, led by our Information Specialist (AB), through examination of key studies and discussion. It captured the concepts of school gardening and horticulture activities. The following MeSH terms were used: school exp; gardening exp; child nutrition sciences. No methods filters were used. The master search strategy (Table 1) was adapted and run in the following electronic databases in February 2014 and updated in May 2015: MEDLINE, EMBASE, PsycINFO, HMIC and SPP (using the OVID interface); AEI, BEI, ASSIA, BNI 1994-current and ERIC (using the ProQuest interface); AMED and CINAHL (using the EBSCOHost interface). Additional grey literature databases were also searched: OpenGrey, EThOS and British Library Catalogue. The review by the National Foundation for Education Research was a useful source of includable references [8]. Reference lists of included studies were scrutinised for other relevant studies. Forward citation searches were undertaken on included studies. Citation searches were also performed in Web of Science using three key references [15–17].

**Table 1:** Search strategy for the health and well-being impacts of school gardening (as used in Medline)

1	school*.tw.
2	educat*.tw.
3	garden*.tw.
4	horticult*.tw.
5	(horticult* adj3 (school* or educat*)).tw.
6	(Food or fruit* or vegetable*).tw.
7	((Food or fruit* or vegetable*) adj2 grow*).tw.
8	((Food or fruit* or vegetable*) adj2 production).tw.
9	((Food or fruit* or vegetable*) adj2 producing).tw.
10	((Food or fruit* or vegetable*) adj2 plant*).tw.
11	7 or 8 or 9 or 10
12	exp Schools/
13	exp Gardening/
14	*"Child Nutrition Sciences"/
15	1 or 12
16	3 or 13 or 14
17	15 and 16
18	11 and 15
19	(educat* adj3 garden*).tw.
20	17 or 18 or 19

### Inclusion criteria

Studies were considered eligible for inclusion if they met the following criteria:

- *Population*: School children, school staff, family and community members (all ages) were included. Studies conducted in OECD countries and published in English were included.
- *Interventions*: Studies were included if they reported the effects of participation in school gardening activities. The definition of ‘school’ included all educational settings up to 18 years, including special schools. The definition of ‘gardening’ included growing or cultivating any kind of plants (such as vegetables, fruits, trees, shrubs and flowers). Gardening activities included preparing the soil, planting, weeding, watering, harvesting and garden-related cooking and tasting activities. These gardening activities were either integrated into the curriculum, or conducted outside of lesson time (e.g. lunchtime clubs, after school clubs, school-organised trips to community allotments). Gardening activities for school-age children that did not involve schools were not included (e.g. summer holiday clubs or community youth interventions).
- *Comparators:* Quantitative studies were only included if groups participating in school gardening activities were compared with control groups or groups participating in alternative activities (such as nutrition education without gardening activities). This criterion was not relevant for qualitative studies.
- *Outcomes:* Studies were included if they reported quantitative or qualitative health and well-being outcomes including dietary intake; food-related knowledge, attitudes and preferences; physical, mental or emotional health; quality of life indicators. Qualitative findings also included themes, concepts and metaphors relating to the experience and meaning of school gardens, and any perceived factors that help or hinder their success. Additional outcomes, including adverse or unintended outcomes, were only considered where they were reported alongside health and well-being outcomes.
- *Study design*: Suitable quantitative study designs included randomised controlled trials (RCT), non-randomised controlled trials, and other ‘controlled before and after’ studies. Suitable qualitative study designs included any recognised methods of data collection and analysis from any discipline or theoretical tradition. The types of data collection methods included (but were not limited to): focus groups, individual interviews, participant or systematic observation, documentary analysis, audio/visual/note collection. Methods of analysis included (but were not limited to): grounded theory, narrative analysis, thematic analysis, phenomenological analysis, discourse analysis.

### Selection process

References identified through the search strategy were uploaded into ENDNOTE (X7, Thomson Reuters). References (titles/abstracts) were independently double screened using the eligibility criteria (by reviewers HO/RW/SG). Studies appearing to meet the criteria were obtained as full text articles. Full texts were independently double screened using the same criteria (by reviewers HO/RW/SG/RG). Any disagreements were resolved through discussion with the whole team.

During full text screening, additional inclusion criteria were developed as an iterative process. For example, studies in which school gardening was one of multiple components in a ‘whole school approach’ intervention (in combination with farm visits, school food policy development, school meals and catering reforms, nutrition education or cookery programmes) were not included if the reported outcomes were not specifically attributable to school gardening. Some qualitative studies did report health and well-being impacts attributable to school gardening (i.e. distinct from other components) and these studies were included. Studies that did not report sufficient information about data collection and/or analysis methods to enable critical appraisal were excluded (such as conference abstracts for which the full text was not available).

### Data extraction

Standardised, piloted data extraction sheets were developed for the review to ensure consistency between studies and between reviewers. The data extracted for each study included, where possible: study design, sample characteristics, description of school gardening activities (intervention group), description of alternative activities (comparison group), duration of intervention, data collection methods, analysis methods and duration of follow up. For quantitative studies, health and well-being outcomes (and other secondary outcomes) were extracted. For qualitative studies, findings in the form of participants’ quotes and author themes and concepts were extracted. Data were extracted by one reviewer (HO) and independently double checked (by reviewers RW/SG/RG). Any disagreements were resolved through discussion with the whole team.

### Quality appraisal

The quality of included quantitative studies was appraised using the Effective Public Health Practice Project (EPHPP) Quality Assessment Tool for Quantitative Studies. Each study was rated strong, moderate or weak for the following components: selection bias, study design, confounders, blinding, data collection, withdrawals and drop outs. A ‘global’ or overall rating was then allocated using the standard system: strong (no weak ratings), moderate (one weak rating), or weak (two or more weak ratings).

The quality of included qualitative studies was appraised using criteria suggested by Wallace et al. [18]. In addition to the standard ratings of yes, no, can’t tell, we used ‘partial’ in some cases, for example studies in which some ethical issues were addressed and others were not (Wallace criterion 12). We also generated overall quality ratings using our own system: strong (11–12 ratings of yes); moderate (6–10 ratings of yes); weak (1–5 ratings of yes).

Studies were appraised by one reviewer (HO) and independently double checked (by reviewers RW/SG/RG). Any disagreements were resolved through discussion with the whole team.

### Data synthesis

It was not possible to meta-analyse any of the quantitative data we extracted due to study design and data limitations (further information provided in Tables). Authors were contacted to clarify some study details (such as precise methods of dietary assessment) before this decision was confirmed. Data from quantitative studies were therefore tabulated, grouped by similar outcomes and the effectiveness of the school gardens in influencing health and well-being outcomes was described narratively.

The qualitative data were synthesised thematically in order to understand the perceived well-being and wider impacts of gardens, as well as factors influencing success and sustainability, in order to make practical recommendations for interventions [14, 19]. Narrative synthesis has three overlapping stages: 1) coding of the findings of primary qualitative studies; 2) organising codes into descriptive themes and concepts; 3) generating analytical themes – this final stage goes beyond the interpretations of the original studies and aims to generate new understandings or hypotheses in relation to the review questions [14]. Initially, all study findings were coded, including those that did not relate directly to health and well-being impacts. Where multiple publications reported findings from the same qualitative study, we were careful not to ‘double count’ findings. Therefore, where multiple publications by the same team of authors reported similar themes, only one publication has been referenced for each theme (the oldest one). In stages 1 and 2, we used an inductive approach to code the data (line by line) and identify common themes and categories of themes between studies. This was done manually rather than using software, using the principles of constant comparison and reciprocal translation [20]. Care was taken to recognise divergent data and to interpret the raw data (where presented) rather than uncritically accept the authors’ interpretations. In stage 3, we developed a conceptual model to summarise and illustrate proposed mechanisms for the health and well-being impacts of school gardening. The qualitative synthesis was completed by two reviewers (HO/SG) in discussion with the rest of the team. Throughout the synthesis, we considered study quality, context and transferability as we developed our conceptual model.

## Results

### Search results

**Fig. 1:**
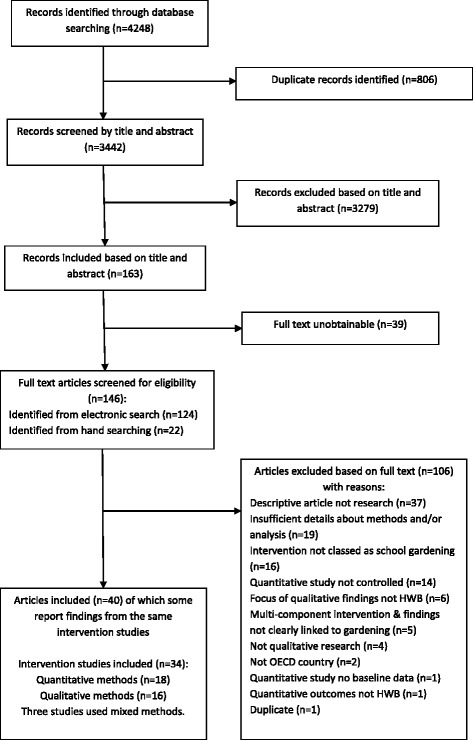
Flow chart showing the identification and selection of studies

Electronic searches identified a total of 3442 records after the removal of duplicates. Title and abstract screening resulted in the exclusion of 3279 records that did not meet the inclusion criteria. 124 of the remaining potentially relevant articles were obtained in full text format (whilst 39 were unobtainable) and another 22 eligible articles were found by citation and manual searching. Full text screening resulted in the exclusion of 106 articles (Fig. 1). A total of 40 articles (hereafter referred to as studies) were eligible for inclusion.

### Study characteristics

The 40 included studies were from the UK, Portugal, USA and Australia. Twenty four studies (including three that were mixed methods studies) reported quantitative methods and findings (Table 2). There was some duplication where multiple publications reported findings from the same trials, which were: five cluster randomised controlled trials (RCT) [9, 10, 21–23] and 13 non-randomised controlled trials [15–17, 24–39] (Table 2). Nineteen studies (including three that were mixed methods studies) reported qualitative methods and findings using a variety of study designs which met our inclusion criteria [11–13, 24–26, 40–52] (Table 3). Again there was some duplication where multiple publications reported findings from the same sample (Table 3).

**Table 2:** Summary of included quantitative studies (*n* = 18)

First author (year) Publication type	Study design	Country	Type of schools	Sample size (baseline)	Sample characteristics	Intervention group (duration)	Comparison or control group	Outcomes (health and well-being only)
Block (2012)^a^ [24] Journal paper	Non-randomised controlled	Australia	Primary	764 children (reported as 770 in Block et al. 2009) 562 parents	8–12 years 54 % girls	Stephanie Alexander Kitchen Garden Program (45–60 min in garden class & 90 min in kitchen class/week for 12+ mnths)	No intervention (but Gibbs et al. reported that some children were exposed to some gardening and cooking activities)	Child quality of life
Block (2009)^a^ [25] Report								Willingness to try new foods
Gibbs (2013)^a^ [26] Journal paper								Willingness to try new foods Food and beverage intakes including FV
Brouwer (2013) [27] Journal paper	Cluster RCT	USA	Pre-school	12 children	3–5 years	Watch Me Grow (weekly activities for four months)	No intervention (delayed)	FV served and consumed
Christian (2014) (1) [28] Journal paper: Trial 1	Cluster RCT	UK	Primary	1138 children (reported as 1256 in the journal paper)	For two groups respectively: Mean 8.2/8.1 year 50/51 % boys 30/35 % White British (diverse)	Royal Horticultural Society (RHS) led gardening activities (18 months with regular support visits and termly teacher training sessions from RHS)	Teacher led gardening activities (18 months with termly teacher training sessions from RHS)	Vegetable intake Fruit intake Food group and essential nutrient intakes
Christian (2014) (2) [9] Report: Trial 1								As above plus: FV knowledge Attitudes towards FV
Christian (2014) (2) [10] Report: Trial 2	Cluster RCT	UK	Primary	1391 children	For two groups respectively: Mean 8.3/8.2 years 52/48 % boys 23/17 % White British (diverse)	Teacher led gardening activities (15 months with termly teacher training sessions from RHS)	No intervention	Vegetable intake Fruit intake Food group and essential nutrient intakes FV knowledge Attitudes towards FV
Cotter (2013) [22] Journal paper	Cluster RCT	Portugal	NR	155	10–12 years	Aromas school gardening club (2 h/week for 6 months) plus regular lectures on the dangers of high salt intake	Regular lectures on the dangers of high salt intake	Body Mass Index (BMI) Waist circumference Blood pressure (SBP/DBP) Urinary sodium Urinary creatinine Estimated salt intake
Wells (2014) [23] Journal paper	Cluster RCT	USA	Elementary	285	8–12 years	Healthy Gardens, Healthy Youth pilot program: gardening activities plus curriculum of 20 lessons (1 year)	No intervention (control group received gardens at the end of the study)	Physical activity
Cotugna (2012) [27] Journal paper	Non-randomised controlled	USA	Elementary	359	Age or gender not reported; For A/B/C respectively: 73/41/37 % White 37/34/38 % low income	Gardening education program (duration unknown) first time in School B and second time in School C	No intervention (School A)	Students who chose salad for lunch
Davis (2011) [28] Journal paper	Non-randomised controlled	USA	Elementary	107 (reported as 104 in Davis et al. 2011)	9–11 years 59 % overweight or obese For two groups respectively: Mean 9.7/9.9 years 38/59 % boys 97/93 % Latino	LA Sprouts: cooking and nutrition lessons plus gardening activities (90 min per week for 12 weeks)	No intervention	Body Mass Index (BMI) Waist circumference Total body fat Blood pressure (SBP/DBP) Vegetable intake Fruit intake Food group and macronutrient intakes
Gatto (2012) [29] Journal paper								Motivation to eat FV Attitudes, preferences and perceptions relating to cooking FV
Jaenke (2012) [15] Journal paper	Non-randomised controlled	Australia	Primary	127	Fifth and sixth grade students 11–12 years 54 % boys	Nutrition education: How do you grow? (3 h over 10 weeks) plus gardening: How does your garden grow? (180 min per week for 10 weeks)	Nutrition education only: How do you grow? (3 h over 10 weeks) No intervention	Willingness to taste vegetables Taste ratings of vegetables Fruit intake Vegetable intake
Morgan (2010) [30] Journal paper								Fruit intake Vegetable intake Ability to identify vegetables Willingness to taste vegetables Taste ratings of vegetables FV knowledge Quality of school life
McAleese (2007) [16] Journal paper	Non-randomised controlled	USA	Elementary	122	10–13 years Mean 11.1 years 44 % boys	Nutrition education: Nutrition in the garden, plus gardening (12 weeks)	Nutrition education only: Nutrition in the garden (12 weeks) No intervention	Fruit intake Vegetable intake Vitamin A intake Vitamin C intake Fibre intake
Meinen (2012) [31] Journal paper	Non-randomised controlled	USA	Elementary schools and early childhood sites	404 youth 567 parents	7–13 years 54 % boys For two groups respectively: Mean 9.9/10.1 years Majority/88 % White	Youth gardening program: Got Dirt? (4 months)	No intervention	Willingness to try new FV Like/dislike of FV Knowledge of FV FV consumption
Morris (2001) [32] Journal paper	Non-randomised controlled	USA	Elementary	97	First grade students	Nutrition education plus gardening (8 months)	No intervention	Nutrition knowledge Willingness to taste vegetables Taste ratings of vegetables
Morris (2002) (1) [33] Journal paper	Non-randomised controlled	USA	Upper elementary	215 (reported as 213 in journal paper)	9–10 years 8.4 % African American 3.0 % Asian American 17.2 % Hispanic 66.5 % White	In-class nutrition education including hands-on gardening activities (9 lessons over 17 weeks)	In-class nutrition education only (9 lessons over 17 weeks) No intervention	Nutrition knowledge Vegetable preference
Morris (2002) (2) [34] Report								
O’Brien (2006) [35] Journal paper	Non-randomised controlled	USA	Elementary	38	9–10 years 50 % boys 71 % White	After school gardening club (8 lessons with 30 min gardening over 10 weeks)	No intervention	Nutrition knowledge FV preference FV consumption self-efficacy FV consumption expectations
Parmer (2009) [37] Journal paper	Non-randomised controlled	USA	Elementary	115	70 % boys For three groups mean respectively: 7.3/7.5/7.4 years 46/27/28 % girls	Nutrition education plus gardening (1 h alternating nutrition education and gardening for 28 weeks)	Nutrition education only (1 h every other week for 28 weeks) No intervention	FV knowledge FV preferences FV consumption
Parmer (2007) [36] Dissertation								
Ratcliffe (2011) [38] Journal paper	Non-randomised controlled	USA	Middle	320	11–13 years 22 % African American 29 % Asian American 9 % Filipino American 30 % Latino 3 % Pacific Islander 7 % White or other 35 % overweight 64 % low income	Garden-based learning activities integrated into science class (20 min instruction and 40 min hands-on gardening per week for 4 months)	Covered the same health and science objectives but did not include a gardening program	Vegetable knowledge Vegetables preferences Willingness to taste Vegetable consumption
Robinson (2005) [39] Journal paper	Non-randomised controlled	USA	Elementary	281	Third, fourth and fifth grade students (no further info)	School gardening curriculum: Texas Agricultural Extension Service (varied intensity over one school year)	No intervention (until after study period)	Life skills: working with groups; self-understanding; leadership; decision making; communication; volunteerism
Waliczek (2001) [17] Journal paper	Non-randomised controlled	USA	Elementary and junior high	589	8–15 years 43 % boys at post-test	Project GREEN school garden program (Spring semester)	No intervention	Interpersonal relationships

^a^also included for qualitative findings (see Table 3); *FV* fruits and vegetables

*FV* fruits and vegetables, *SBP* systolic blood pressure, *DBP* diastolic blood pressure

**Table 3:** Summary of included qualitative studies (*n* = 16)

First author (year)	Country	Sample characteristics	Aims	Sampling methods	Intervention	Data collection methods Analysis methods
Ahmed (2011) [40]	USA	Administrators (*n* = 2), teachers (*n* = 4) and garden staff (*n* = 3) at one rural middle school; school population 50 % Native Hawaiian; low socio-economic status	To examine perceptions of educators about the effects of school-based gardens on children's health and obesity	Snowball sampling starting with the school principle and garden leader	School garden program founded to prevent nutrition-related illness (with community involvement)	Semi structured interviews (4 years after garden established) Grounded Theory approach using descriptive, open coding; list of themes used to develop a conceptual model
Alexander (1995) [41]	USA	Students (*n* = 52), teachers (*n* = 5), parents (*n* = 3), principal and Master Gardener at one inner city elementary school; students 70 % Hispanic; many from single parent homes	To identify the effects on school children participating in classroom gardens	NR	Master Gardeners’ Classroom Garden Project	Interviews (individual and group) and observation Constant comparative method; multiple sources of data evaluated for emerging themes
Anderson (2011) [42]	USA	Students (*n* = 14) at one rural high school	To determine the impact of hydroponically grown vegetables on obesity indices	Purposely selected students twice during the two-year project	Hydroponic gardening system	Focus groups (*n* = 7 at each time point i.e. twice during the two-year project)
Block (2012)^a^ [24]	Australia	Six program schools and six comparison schools; all primary At program schools only: classroom teachers (*n* = 26), volunteers (*n* = 17), other parents (*n* = 20), children (*n* = 124), kitchen and garden specialist staff (*n* = 10) At all participating program and comparison schools: school principals (*n* = 12)	To explore participants' expectations and experiences of the program, changes in the school and home environment, highlights and areas for potential improvement	Convenience sampling (all adults invited to participate) and purposive sampling (teachers selected children with range of ages and program experience)	Stephanie Alexander Kitchen Garden Program	Focus groups, individual interviews, participant observation, field notes and researcher reflections (at various time points before, during and after the program) Inductive thematic content analysis to identify emerging themes and patterns, which were then further analysed according to their relationship with the existing evidence base and theoretical perspectives
Block (2009)^a^ [25]						
Gibbs (2013)^a^ [26]			To evaluate the achievement of the program in increasing child appreciation of diverse, healthy food			
Townsend (2014) [43]			To explore motivations for and impacts of volunteering with the gardening program			
Bowker (2007) [44]	UK	Two classes from one primary school and one secondary school; 7–14 years	To gain an understanding of what the children themselves think about school gardening	Quota sampling to identify two schools; within each school a class unit was selected to further refine the sample; 12 children in each class were randomly selected for interviews	Gardens for Life (to support and extend learning in other curriculum areas)	Concept maps (*n* = 72) supported by contextual observation, semi-structured interviews (*n* = 24) (after 6 months) and children’s drawings Interpretive approach - broad concepts were identified and organised into categories; concept grids and depth scores used to look for patterns
Chawla (2014) [11]	USA	Students (*n* = 52), teachers and school principals from four high schools; students 14–19 years; 60 % girls; European-American (*n* = 29); Hispanic (*n* = 19); Asian (*n* = 3); Pacific Islander (*n* = 1)	Research questions: How do students experience natural areas on their school grounds? What values do students find in these natural areas?	Purposive sampling to span the high school age range	Four different gardening programs at four high schools: gardening as school service (elected); agricultural biology class (elected); horticultural science class for teen mothers (required); after school and summer gardening program (voluntary)	Ethnographic observations recorded through field notes, video or photography, and open-ended, semi-structured interviews Data was repeatedly reviewed with attention to repetitive refrains, recurring patterns and resonant metaphors; triangulation of methods to identify similar themes and discordant data
Chiumento (2012) [12]	UK	Students (*n* = 36) with signs of Behavioural, Emotional & Social Difficulties (BESD) from two primary and one secondary schools; 10–15 years; 61 % boys; mix of nationalities and ethnicities including children seeking asylum; deprived ward in Liverpool	NR	Students were referred by schools, providing pen profiles of current difficulties including potential behavioural risk factors	Haven of Greenspace (social and therapeutic horticulture); pupil led sessions using NFER five ways to well-being framework (monthly for 6 months)	Draw and write journals (children); closing semi-structured interviews (link teachers); reflective process diary by group therapists Thematic analysis of interview transcripts; random selection of journals analysed with quality checks
Cutter-Macenzie (2009) [45]	Australia	Students (*n* = ?) from one city primary school; 6–12 years; all students participating in program (*n* = 70) had English as a second language and some were recent migrants	To assess the impact of the program against its objectives which included helping to develop strong local communities and school communities; and fostering healthy eating habits	NR	Multicultural school gardens program created to enable disadvantaged schools to establish a culturally focused gardening program (2 years)	Children as researchers including journals, photographs and peer interviews (*n* = 10); researcher’s field visits, observations and interviews with children and teachers (after 3 months)
Hazzard (2011) [46]	USA	Administrators, teachers, parent and community volunteers and garden coordinators (*n* = ?) from 10 schools (elementary, middle and high schools)	To ascertain and report best practices for schools implementing or sustaining instructional school gardens	Stratified random sampling from list of all schools with exemplary instructional school gardens programs; principals selected individuals directly involved with the success of the gardens	California Instructional School Garden Program (CISGP)	Interviews with key members Constant comparative analysis; results used to create best practice models for schools in California and across the United States
Henryks (2011) [47]	Australia	Parents of children enrolled at the school (*n* = 5) and another member of the wider community (*n* = 1) at one primary school	To explore the role played by the school kitchen garden in the lives of its associated volunteers	Purposive sampling by email invitation to volunteers	Stephanie Alexander Kitchen Garden Program	In-depth interviews Thematic analysis used to build a conceptual map of the experiences of the school kitchen garden volunteers, including the motivations, benefits and challenges that volunteers experienced; combination of inductive and deductive approaches
Lakin (2008) [48]	UK	Head teacher, a governor, a teacher and groups of children in Year 3 (*n* = 5) and Year 6 (*n* = 5) at one semi-rural primary school; 7–11 years	NR	School B selected to represent example of good practice; children selected by the head teacher for their involvement in the innovations	Health Promoting Schools: Gloucestershire Food Strategy	Detailed interviews; observations; classroom display and classroom activities as exemplified by the children's workbooks (over 3 days of visits)
Miller (2007) [49]	USA	Teachers (*n* = 19) and children (*n* = ?) from one early education setting: Dimensions Educational Research Foundation; 3–6 years	To examine the skills young children are developing when they are engaged in developmentally appropriate activities in the greenhouse and garden	NR	Dimensions outdoor classroom including garden and greenhouse areas (two small group activities a month)	Teachers’ documentation (nature notes) of children interacting with nature in the garden/greenhouse; children’s drawings and work from their garden/greenhouse experiences (*n* = ?); focus group interviews conducted with teachers (*n* = 19) on three occasions over two years. Teachers’ nature notes and children’s work were analysed using a systematic framework from prior data analysis of teachers’ visual notes; key themes identified from focus groups
Ming Wei (2012) [13]	USA	Students (*n* = 20), teachers (*n* = 9), school principal, school counsellor, student services coordinator and parents/caregivers (*n* = 4) from one rural elementary school; students 55 % girls; from low to middle income families; native culture	To better understand the experience of student learning in the context of school garden-based education and to determine the relevance of school gardens as a site for learning making	Convenience sample of third, fourth and fifth grade Gifted and Talented students who spent two or more hours in the garden each week	The Discovery Garden: using an interdisciplinary standards-based school garden curriculum	Formal interviews and talk story (informal chats); field notes collected during the garden classes and garden-based activities (over one semester) Listened and looked for recurring patterns; constructed of a network of related and connected themes; content analysis using constant comparative methods
Passy (2010) [50]	UK	Two samples (two stages) from 10 primary schools e.g. stage 1: senior leaders (*n* = 11), garden leads (*n* = 10), other members of teaching staff (*n* = 10), teaching assistants (*n* = 2), parent governors (*n* = 2), other parents (*n* = 2) and pupils (*n* = 43)	To assess the impact that using a school garden had on primary pupils’ learning, behaviour and health and wellbeing	Stratified random sampling from list of participating schools; weighted towards those with higher levels of benchmark achievement	Campaign for School Gardening (Royal Horticultural Society)	Case studies including interviews and observations (two stages over six months); schools were given disposable cameras and diaries in which to record activities
Somerset (2005) [51]	Australia	Teachers responsible for vegetable gardens at 12 primary schools	To investigate the nature and extent of the use of school gardens in a defined region of eastern Australia	All schools with vegetable gardens (outdoor or greenhouse) as identified by telephone survey	Schools with vegetable gardens (no one intervention)	Open ended questionnaire; face-to-face interviews Data were then categorised thematically and analysed
Viola (2006) [52]	Australia	Key informants from one primary school (*n* = 6) and one secondary school (*n* = 9); students in grades 4–9; Indigenous Australians; remote rural communities	To examine how effective school gardens are as a nutritional education tool in Indigenous Australian school settings	Schools selected by researcher; participating grades determined by school principals; key informants selected from each community advisory group	Outreach School Garden Project (incorporated formal nutrition and gardening education lessons into the core school curriculum	Semi-structured interviews; reflective journal; event log (over six months with outreach visits for 3–5 days every 6–8 weeks) Descriptive qualitative approach; triangulation of research methods and data sources

^a^also included for quantitative findings (see Table 2)

Only two school gardening interventions, the RHS Campaign for School Gardening and the Stephanie Alexander Kitchen Program, generated quantitative and qualitative evidence for the same intervention (Tables 2, 3 and 4) [9, 10, 24–26, 50]. The studies were conducted in pre-schools [21, 31, 49], primary schools [9, 10, 12, 15, 24–26, 30, 43–45, 47, 48, 50–52], elementary schools [13, 16, 17, 23, 27–29, 31, 32, 35–37, 39, 41, 46], upper elementary schools [33, 34], middle schools [38, 40, 46], junior high schools [17], high schools [11, 42, 46] and secondary schools [12, 44, 52] (Tables 2 and 3). The school gardening interventions included a variety of components and characteristics (Table 4). For example, some were purely gardening interventions [9–13, 17, 21, 27, 31, 39–42, 44, 46, 49–51], whereas others incorporated cooking and/or nutrition education alongside the gardening activities [15, 16, 22–26, 28–30, 32–38, 43, 45, 47, 48, 52].

**Table 4:** Components and characteristics of school gardening interventions included in this review

First author (year) Multiple studies about the same intervention are grouped together.	Name of school gardening intervention	Gardening component	Cooking as key component	Nutrition education component	Integrated with wider curriculum	Produce used in school catering	Outdoors some or all of the time	Delivered by specialists	Delivered by teachers	Teachers trained by specialists	Community involvement	Theory-led intervention
Ahmed (2011) [40]	No name (school garden program founded to prevent nutrition-related illness)	✓			✓						✓	
Alexander (1995) [41]	Master Gardeners’ Classroom Garden Project	✓						✓	✓	✓		✓
Anderson (2011)	Hyrdoponic gardening system	✓			✓						✓	
Block (2012) [24] Block (2009) [25] Gibbs (2013) [26] Henryks (2011) [47] Townsend (2014) [43]	Stephanie Alexander Kitchen Garden Program	✓	✓		✓		✓	✓	✓		✓	✓
Bowker (2007) [44]	Gardens for Life	✓			✓						✓	✓
Brouwer (2013) [21]	Watch Me Grow	✓			✓	✓	✓		✓	✓	✓	
Chawla (2014) [11]	No name (four different gardening programs at four high schools)	✓					✓		✓			✓
Chiumento (2012) [12]	Haven of Greenspace (social and therapeutic horticulture)	✓					✓	✓				✓
Christian (2014) (1), [9] Christian (2014) (2) [10] Passy (2010) [50]	Royal Horticultural Society (RHS) Campaign for School Gardening	✓			✓		✓	✓	✓	✓	✓	✓
Cotter (2013) [22]	Aromas school gardening club	✓		✓			✓					
Cotugna (2012) [27]	Gardening education program	✓					✓					
Cutter-Macenzie (2009) [45]	Multicultural School Gardens Program	✓	✓		✓		✓		✓		✓	
Davis (2011) [28] Gatto (2012) [29]	LA Sprouts	✓	✓	✓			✓	✓	✓	✓	✓	✓
Hazzard (2011) [46]	California Instructional School Garden Program	✓			✓						✓	
Jaenke (2012) [15] Morgan (2010) [30]	How do you grow?/How does your garden grow?	✓	✓	✓	✓	✓	✓		✓	✓	✓	✓
Lakin (2008) [48]	Health Promoting Schools: Gloucestershire Food Strategy	✓	✓	✓	✓	✓	✓				✓	
McAleese (2007) [16]	Nutrition in the garden	✓	✓	✓			✓					
Meinen (2012) [31]	Youth gardening program: Got Dirt?	✓							✓	✓		
Miller (2007) [49]	Dimensions outdoor classroom including garden and greenhouse areas	✓			✓		✓		✓		✓	
Ming Wei (2012) [13]	The Discovery Garden	✓			✓		✓		✓			✓
Morris (2001) [32] Morris (2002) (1) [33] Morris (2002) (2) [34]	No name (nutrition education plus gardening)	✓	✓	✓	✓	✓	✓		✓		✓	✓
O’Brien (2006) [35]	No name (based on Junior Master Gardener)	✓		✓			✓	✓	✓			
Parmer (2009) [37] Parmer (2007) [36]	No name (based on Pyramid Café/Health and Nutrition from the Garden)	✓	✓	✓	✓		✓		✓			✓
Ratcliffe (2011) [38]	No name (garden-based learning activities)	✓	✓	✓	✓	✓	✓		✓		✓	✓
Robinson (2005) [39]	No name (school gardening curriculum)	✓			✓		✓		✓	✓	✓	
Somerset (2005) [51]	No name (schools with vegetable gardens)	✓					✓					
Viola (2006) [52]	Outreach School Garden Project	✓		✓	✓				✓			✓
Waliczek (2001) [17]	Project GREEN school garden program	✓			✓				✓			
Wells (2014) [23]	Healthy Gardens, Healthy Youth	✓		✓	✓		✓		✓			

Note: some studies did not report sufficient details about the intervention, so no tick may mean not applicable or not reported

### Duplication and differences in reporting

Where multiple publications duplicated findings from the same trials, we have been very careful to present and synthesise the findings of these studies without duplication; for example elements of Morgan’s findings were re-published by Jaenke [15, 30]). To confuse matters, differences were found in the reporting of the same outcomes between different studies. For example, Jaenke and Morgan both reported data from a school garden-based nutrition education program in Australia (*n* = 127) but they reported slightly different findings in the control group for fruit and vegetable intake [15, 30]. Neither of these reported findings were statistically significant and we have presented the most recent findings [15] (Table 7). Another case of duplication and differences in reporting was in the evaluation of the pilot of the Stephanie Alexander Kitchen Garden Program in Australia [25, 26]. Both studies reported children’s willingness to try new foods, but with slightly different sample sizes (*n* = 770/*n* = 764) and therefore slightly different findings. We have presented the findings from the original study which also reported the largest sample size [25] (Table 9). As illustrated, in cases such as these, the data from the study reporting the largest sample size (or, if the sample sizes were equal, the most recent study) were presented in our review tables.

### Quality appraisal of included studies

**Fig. 2:**
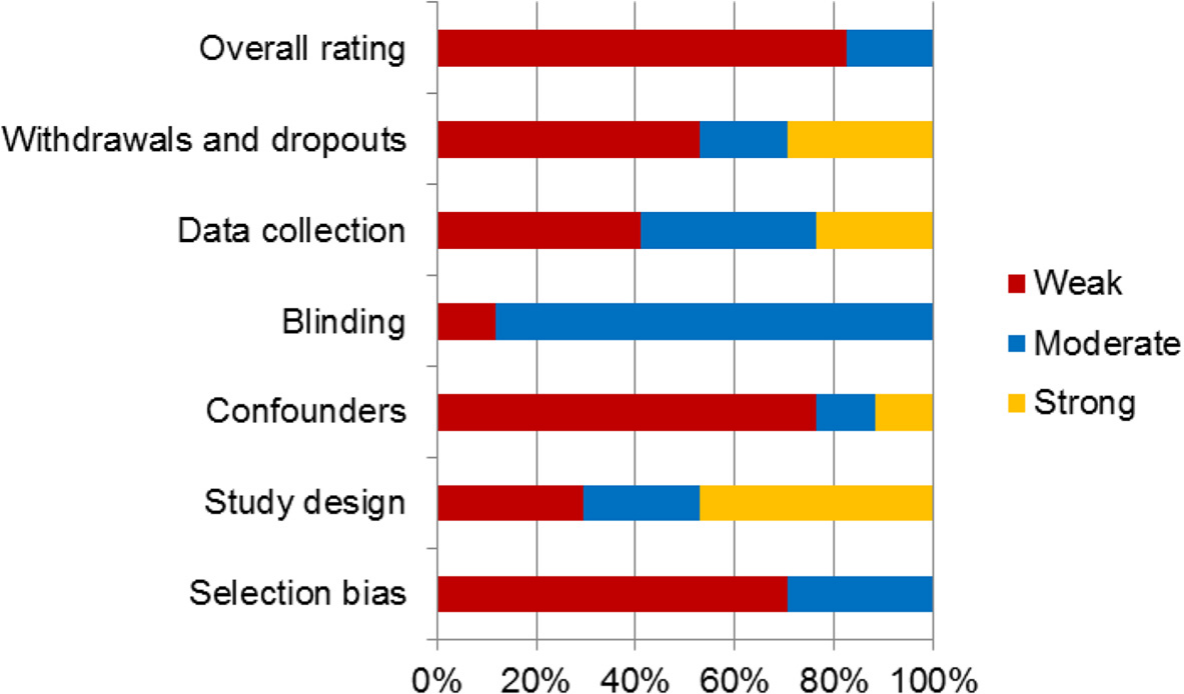
Graph showing quality ratings of included quantitative studies (*n* = 24) using the individual EPHPP criteria and overall rating

Where multiple studies reported quantitative data from the same trial (quantitative) or the same sample (qualitative), they were appraised collectively (Tables 5 and 6). Most of the quantitative studies were rated as weak, with six studies rated as moderate [10, 15, 23, 28–30] (Table 5). Quality criteria that were commonly rated weak (in more than half the studies) using the EPHPP system were selection bias, control for confounders, and follow-up rate (withdrawals and drop-outs) (Fig. 2). While five qualitative studies were rated as strong, most of the qualitative studies were rated as weak or moderate quality [11, 24–26, 43] (Table 6). This reflects the often unclear reporting about some aspects of quality in qualitative studies, such as theoretical perspective, adequacy of sample and selection methods, data collection methods, analysis methods, consideration of limitations and of ethical issues.

**Table 5:** Quality appraisal of included quantitative studies (*n* = 18)

First author (year)	EPHPP criteria for quantitative studies						
	Selection bias	Study design	Confounders	Blinding	Data collection	Withdrawals and dropouts	Overall rating
Block (2012) [24] Block (2009) [25] Gibbs (2013) [26]	Weak	Strong	Weak	Moderate	Moderate	Strong	Weak
Brouwer (2013) [21]	Weak	Strong	Weak	Moderate	Strong	Weak	Weak
Christian (2014) (1), [9] Christian (2014) (2) Trial 1 [10]	Weak	Strong	Moderate	Moderate	Moderate	Weak	Weak
Christian (2014) (2) Trial 2 [10]	Weak	Strong	Moderate	Moderate	Moderate	Moderate	Moderate
Cotter (2012) [22]	Weak	Strong	Weak	Moderate	Strong	Strong	Weak
Cotugna (2012) [27]	Moderate	Strong	Weak	Weak	Weak	Weak	Weak
Davis (2011) [28] Gatto (2012) [29]	Moderate	Strong	Strong	Moderate	Strong	Strong	Moderate
Jaenke (2012) [15] Morgan (2010) [30]	Moderate	Moderate	Strong	Moderate	Weak	Strong	Moderate
McAleese (2007) [16]	Weak	Strong	Weak	Moderate	Moderate	Weak	Weak
Meinen (2012) [31]	Moderate	Moderate	Weak	Weak	Weak	Moderate	Weak
Morris (2001) [32]	Weak	Moderate	Weak	Moderate	Weak	Weak	Weak
Morris (2002) (1) [33] Morris (2002) (2) [34]	Moderate	Weak	Weak	Moderate	Moderate	Strong	Weak
O’Brien (2006) [35]	Weak	Weak	Weak	Moderate	Weak	Weak	Weak
Parmer (2007) [36] Parmer (2009) [37]	Weak	Moderate	Weak	Moderate	Weak	Weak	Weak
Ratcliffe (2011) [38]	Weak	Weak	Weak	Moderate	Moderate	Moderate	Weak
Robinson (2005) [39]	Weak	Weak	Weak	Moderate	Weak	Weak	Weak
Waliczek (2001) [17]	Weak	Weak	Weak	Moderate	Strong	Weak	Weak
Wells (2014) [23]	Weak	Moderate	Moderate	Moderate	Moderate	Moderate	Moderate

Where multiple publications reported quantitative data from the same study, they were appraised as one study

**Table 6:** Quality appraisal of included qualitative studies (*n* = 16)

First author (year)	Wallace criteria													Total # Yes ratings	Overall rating
	1	2	2b	3	4	5	6	7	8	9	10	11	12		
Ahmed (2011) [40]	Y	N	CT	Y	Y	N	Y	Y	Y	Y	Y	Y	Y	10	Moderate
Alexander (1995) [41]	Y	Y	Y	Y	N	P	Y	Y	Y	Y	N	N	CT	8	Moderate
Anderson (2011) [42]	Y	Y	CT	Y	Y	CT	P	P	N	N	P	Y	P	5	Weak
Block (2012) [24] Block (2009) [25] Gibbs (2013) [26] Townsend (2014) [43]	Y	Y	Y	Y	Y	P	Y	Y	Y	Y	Y	NA	Y	11	Strong
Bowker (2007) [44]	Y	Y	Y	Y	Y	P	Y	Y	P	Y	Y	Y	P	10	Moderate
Chawla (2014) [11]	Y	Y	Y	Y	Y	P	Y	Y	Y	Y	P	Y	Y	11	Strong
Chiumento (2012) [12]	Y	P	CT	Y	Y	P	P	P	Y	Y	N	NA	P	5	Weak
Cutter-Mackenzie (2009) [45]	Y	N	CT	Y	Y	P	Y	P	N	Y	Y	Y	P	7	Moderate
Hazzard (2011) [46]	Y	N	CT	Y	P	P	P	P	Y	P	N	N	P	3	Weak
Henryks (2011) [47]	Y	Y	Y	Y	Y	N	Y	P	Y	Y	P	NA	P	8	Moderate
Lakin (2008) [48]	Y	N	CT	Y	Y	P	Y	CT	N	P	N	Y	N	5	Weak
Miller (2007) [49]	Y	N	CT	Y	Y	CT	Y	Y	P	P	N	N	N	5	Weak
Ming Wei (2012) [13]	Y	Y	CT	Y	Y	P	Y	Y	Y	Y	Y	NA	Y	10	Moderate
Passy (2010) [50]	Y	N	CT	Y	Y	Y	Y	CT	N	Y	N	Y	P	7	Moderate
Somerset (2005) [51]	Y	N	CT	Y	N	CT	N	P	P	Y	N	N	CT	3	Weak
Viola (2006) [52]	Y	Y	Y	Y	Y	P	Y	P	N	P	Y	Y	Y	9	Moderate

Where multiple publications reported qualitative data from the same study, they were appraised as one study

*Y* yes, *P* partial, *N* no, *CT* can’t tell, *NA* not applicable

Overall quality rating: strong (11–12 ratings Y); moderate (6–10 ratings Y); weak (1–5 ratings Y)

Legend for Table 6: Wallace criteria (Wallace et al. [18])

1. Is the research question clear?

2. Is the theoretical or ideological perspective of the author (or funder) explicit?

2b. Has this influenced the study design, methods or research findings?

3. Is the study design appropriate to answer the question?

4. Is the context or setting adequately described?

5. Is the sample adequate to explore the range of subjects and settings, and has it been drawn from an appropriate population?

6. Was the data collection adequately described?

7. Was data collection rigorously conducted to ensure confidence in the findings?

8. Was there evidence that the data analysis was rigorously conducted to ensure confidence in the findings?

9. Are the findings substantiated by the data?

10. Has consideration been given to any limitations of the methods or data that may have affected the results?

11. Do any claims to generalisability follow logically and theoretically from the data?

12. Have ethical issues been addressed and confidentiality respected?

The scoring system used above was adapted for the purposes of this review

### Quantitative evidence for the health and well-being impacts of school gardening

#### Fruit and vegetable intakes

Thirteen studies reported children’s fruit and vegetable intakes [9, 10, 15, 16, 21, 26–28, 30, 31, 36–38] (Table 7). Only two studies reported statistically significant increases effects [16, 38]. Both used outcomes based on children’s self-report, and were non-randomised studies that were rated weak in the quality appraisal.

**Table 7:** Results of included quantitative studies: child’s fruit and vegetable intake

First author (year) Sample (*n*)	Outcome measures	Outcomes	Intervention group		Comparison group		Control group		Group x time results (adjusted, if reported)
			Baseline Mean (SD)	Follow-up Mean (SD)	Baseline Mean (SD)	Follow-up Mean (SD)	Baseline Mean (SD)	Follow-up Mean (SD)	
Brouwer (2013) [21] *n* = 12	Structured dietary assessment method for pre-school children (whilst in care)	V served (servings/day) V consumed (servings/day) F served (servings/day) F consumed (servings/day) Serving size = one cup FV; half cup dried fruit; FV juices not included (source: USDA MyPlate)	1.42 (0.67) 0.80 (0.68) 1.55 (0.99) 1.00 (0.89)	1.24 (0.57) 1.05 (0.67) 0.92 (0.56) 0.67 (0.22)	NA	NA	1.13 (0.31) 0.80 (0.38) 0.59 (0.27) 0.32 (0.29)	0.75 (0.21) 0.63 (0.28) 0.49 (0.40) 0.46 (0.43)	NR
Christian (2014) (1) [9] Trial 1; *n* = 1256^†^	CADET (115 items)	F intake (g/day) V intake (g/day) FV intake (g/day)	201 (9.3)^a^ 87 (4.4)^a^ 269 (10.7)^a^	168 (11.8)^a^ 89 (9.0)^a^ 237 (14.5)^a^	214 (9.5)^a^ 102 (4.3)^a^ 300 (10.5)^a^	208 (11.5)^a^ 118 (8.6)^a^ 308 (14.0)^a^	NA	NA	MD = −28 (16.4)^a^; *p* = 0.08 MD = −13 (12.8)^a^; *p* = 0.2 MD = −43 (22.8)^a^; *p* = 0.06
Christian (2014) (2) [10] Trial 1; *n* = 1138^†^									
Christian (2014) (2) [10] Trial 2; *n* = 1391	CADET (115 items)	F intake (g/day) V intake (g/day) FV intake (g/day)	206 (7.9) 95 (3.8) 299 (8.9)	219 (17.6)^a^ 111 (10.2)^a^ 328.8 (23.0)^a^	NA	NA	193 (8.2) 100 (4.7) 296 (9.6)	181 (17.1)^a^ 122 (9.9)^a^ 305 (22.4)^a^	MD = −22 (24.3)^a^; *p* = 0.3 MD = −7 (14.2)^a^; *p* = 0.6 MD = 15 (32.0)^a^; *p* = 0.6
Cotugna (2012) [27] *n* = 359	Lunchtime observations	Students who chose salad for lunch (%)	17.4	24.0	22.2	NR due to scheduling issues	22.1	20.3	NR
Davis (2011) [28] *n* = 104	Block Food Screener (41 items)	F intake (servings/day) V intake (servings/day) Serving size not reported	4.0 (0.7) 1.6 (1.0)	3.9 (0.8) 1.6 (1.0)	NA	NA	4.1 (0.9) 1.9 (1.3)	4.2 (0.8) 1.3 (1.0)	*p* = 0.83 *p* = 0.11
Gibbs (2013) [26] *n* = 764	Parent questionnaire	F ≥ 2 servings/day (%) V ≥ 5 servings/day (%) Serving size = one apple or orange, two kiwis or apricots, one cup dried fruit	84.2 7.7	79.8 7.3	74.6 5.9	72.5 9.5	NA	NA	OR = 1.68 (0.90 to 3.14); *p* = 0.11 OR = 0.87 (0.54 to 1.42); *p* = 0.59
Jaenke (2012) [15] *n* = 127	24 h recall x 2	F intake (servings/day) V intake (servings/day) Serving size = 150 g fruit; 45 g dried fruit; 75 g vegetables	1.2 (1.0) 2.0 (1.7)	Between group mean differences only	1.5 (1.0) 1.9 (1.3)	Between group mean differences only	1.0 (0.9) 2.1 (2.2)	Between group mean differences only	*p* = 0.76 *p* = 0.06
Morgan (2010) [30] *n* = 127 (some differences)^a^									
McAleese (2007) [16] *n* = 122	24 h recall x 3 (workbook)	F intake (servings/day) V intake (servings/day) Serving size not reported	0.8 (0.8) 1.2 (0.6)	1.9 (1.4) 2.6 (1.7)	0.3 (0.5) 1.8 (1.1)	0.5 (0.7) 1.7 (1.0)	0.7 (0.6) 1.7 (0.7)	0.6 (0.7) 1.4 (0.7)	F = 10.98; *p* < 0.001 F = 15.00; *p* < 0.001
Meinen (2012) [31] *n* = 404	Parent survey	Child ate F yesterday (# times) Child ate V yesterday (# times)	2.8 (0.85) 2.5 (0.79)	3.0 (0.88) 2.7 (0.93)	NA	NA	2.8 (0.78) 2.6 (0.79)	2.9 (0.78) 2.6 (0.86)	NR
Parmer (2009) [37] *n* = 115	Lunchroom observations	V consumption (0 = not eaten; 1 = eaten)	0.70 (0.4)	1.0 (0.0)	0.64 (0.5)	0.64 (0.5)	0.83 (0.3)	0.50 (0.5)	NR
Parmer (2007) [36] *n* = 115									
Ratcliffe (2011) [38] *n* = 320	Garden Vegetables Frequency Questionnaire (22 items)	V consumed more than once a month (# varieties)	NR	Change values only reported	NA	NA	NR	Change values only reported	*p* = 0.001
	Taste Test								
		V consumed at school							*p* = 0.010
		V consumed at home							*p* = 0.122

*F* fruits, *V* vegetables, *FV* fruits and vegetables, *SD* standard deviation (or standard error where^a^); *MD* mean difference, *OR* odds ratio, *F* = F statistic from ANOVA

^a^see results text for explanation of how differences in duplicate data reporting were handled

^†^same study but different sample sizes reported

No meta-analysis due to heterogeneity of outcomes (e.g. inconsistent definitions and reporting of FV serving size) and study designs (e.g. different comparison groups; lack of follow-up means; different data collection methods)

#### Nutrient intakes (and other dietary outcomes)

Six studies reported children’s nutrient intakes or other dietary outcomes [9, 10, 16, 22, 26, 28] (Table 8). Four studies reported statistically significant changes in nutrient intake [9, 10, 16, 28]. However, in two studies there was only one statistically significant finding from the multiple nutrient indicators measured, [9, 28] one of which related to a *decrease* in dietary fibre in the control group, rather than improvements in the intervention group [28]. The other study showed more convincing positive effects for three nutrient indicators, but data were based on children’s self-report and was selectively reported for three nutrients only [16].

**Table 8:** Results of included quantitative studies: child’s nutrient intakes (and other dietary outcomes)

First author (year) Sample (*n*)	Outcome measures	Outcomes	Intervention group		Comparison group		Control group		Group x time results (adjusted, if reported)
			Baseline Mean (SD)	Follow-up Mean (SD)	Baseline Mean (SD)	Follow-up Mean (SD)	Baseline Mean (SD)	Follow-up Mean (SD)	
Christian (2014) (1) [9] *n* = 1256^†^	CADET (115 items)	Energy (kcal/day) Protein (g/day) Carbohydrates (g/day) Fibre (g/day) Total fat (g/day) Total sugars (g/day) Iron (μg/day) Sodium (mg/day) Folate (μg/day) Carotene (mg/day) Vitamin C (mg/day)	2034 (39.4)^a^ 75 (1.8)^a^ 265 (4.4)^a^ 13 (0.3)^a^ 82 (2.3)^a^ 132 (2.9)^a^ 11 (0.2)^a^ 2632 (76.3)^a^ 227 (5.3)^a^ 1956 (98.8)^a^ 108 (3.7)^a^	1520 (178.2)^a^ 58 (7.1)^a^ 186 (21.5)^a^ 10 (1.3)^a^ 65 (8.2)^a^ 90 (10.5)^a^ 8 (1.0)^a^ 2272 (286)^a^ 169 (19.7)^a^ 1995 (864)^a^ 113 (31.7)^a^	1993 (34.1)^a^ 73 (1.5)^a^ 267 (4.3)^a^ 13 (0.3)^a^ 78 (1.7)^a^ 134 (2.6)^a^ 11 (0.2)^a^ 2572 (57.6)^a^ 224 (4.5)^a^ 2352 (101.7)^a^ 105 (3.5)^a^	1567 (168.4)^a^ 64 (6.7)^a^ 193 (20.6)^a^ 11 (1.3)^a^ 64 (7.7)^a^ 99 (10.0)^a^ 8 (0.9)^a^ 2257 (267.7)^a^ 180 (18.6)^a^ 2164 (878)^a^ 125 (31)^a^	NA	NA	MD = −46-; *p* = 0.6 MD = −6; *p* = 0.2 MD = −7; *p* = 0.5 MD = −1; *p* = 0.1 MD = 1; *p* = 0.8 MD = −8; *p* = 0.1 MD = −0.4; *p* = 0.5 MD = 16; *p* = 0.9 MD = −11; *p* = 0.3 MD = 168; *p* = 0.5 MD = 13; *p* = 0.02
Christian (2014) (2) [10] Trial 1; *n* = 1138^†^									
Christian (2014) (2) [10] Trial 2; *n* = 1391	CADET (115 items)	Total energy (kcal/day) Fat (g/day) Sodium (mg/day) Total sugars (g/day) Carotene (μg/day) Vitamin C (mg/day) Iron (μg/day) Fibre (g/day) Carbohydrates (g/day) Folate (μg/day) Protein (g/day)	2039 (32.7) 82 (18.0) 2742 (58.4) 133 (2.3) 2024 (74.9) 118 (3.2) 11 (0.2) 13 (0.3) 267 (4.0) 235 (4.5) 75 (1.4)	1845 (172) 76 (7.9) 2621 (259) 108 (11.4) 1841 (299) 75 (30.2) 10 (0.9) 12 (1.2) 227 (21.7) 192 (18.9) 70 (6.5)	NA	NA	1932 (32.8) 78 (2.0) 2575 (64.2) 127 (2.4) 2089 (83.9) 118 (3.2) 11 (0.2) 12 (0.2) 254 (3.6) 220 (4.3) 69 (1.4)	1836 (170) 77 (7.9) 2656 (257) 107 (11.3) 2168 (329) 73 (30) 10 (0.9) 11 (1.2) 225 (21.6) 188 (18.8) 68 (6.4)	MD = 9; *p* = 0.9 MD = −1; *p* = 0.8 MD = −34; *p* = 0.8 MD = 1; *p* = 0.8 MD = −327; *p* = 0.2 MD = 2; *p* = 0.7 MD = 0.1; *p* = 0.8 MD = 0.3; *p* = 0.6 MD = 2; *p* = 0.8 MD = 4; *p* = 0.6 MD = 2; *p* = 0.6
Cotter (2013) [22] * n* = 155	24 h urine samples; flame photometry	Estimated salt intake (g/day)	7.5 (2.4)	6.4 (2.2)	8.1 (3.0)	7.5 (3.0)	7.7 (2.0)	7.4 (3.0)	NR
Davis (2011) [28] *n* = 104	Block Food Screener (41 items)	Energy (kcal/day) Protein (g/day) Fat (g/day) Carbohydrates (g/day) Added sugar (tsp/day) Dietary fibre (g/day) Meat (servings/day) Dairy (servings/day) Whole grains (oz/day)	2011.0 (1410.4) 85.4 (67.7) 79.8 (67.6) 244.2 (145.7) 11.8 (10.2) 16.1 (11.5) 2.1 (2.4) 2.1 (1.3) 0.8 (0.9)	1639.5 (1046.5) 65.1 (43.0) 62.6 (49.6) 211.3 (122.3) 9.9 (9.4) 16.1 (8.6) 2.8 (2.5) 1.7 (1.2) 0.9 (0.7)	NA	NA	1961.0 (1077.5) 81.6 (49.0) 73.3 (52.4) 252.2 (119.6) 11.5 (7.6) 18.7 (10.3) 2.0 (1.7) 2.1 (1.1) 0.7 (0.7)	1535.2 (902.9) 58.3 (38.3) 57.8 (41.4) 202.8 (109.1) 11.2 (9.7) 13.3 (7.5) 2.5 (3.4) 1.7 (1.0) 0.6 (0.6)	*p* = 0.85 *p* = 0.59 *p* = 0.92 *p* = 0.94 *p* = 0.15 *p* = 0.01 *p* = 0.68 *p* = 0.97 *p* = 0.13
Gibbs (2013) [26] *n* = 764	Parent questionnaire	No sweet drinks (%)	74.1	75.6	76.2	68.1	NA	NA	OR = 1.33 (0.70 to 2.5); *p* = 0.38
McAleese (2007) [16] *n* = 122	24 h recall x 3 (workbook)	Vitamin A (μg/day RAE) Vitamin C (mg/day) Fibre (g/day)	430.4 (244.1) 58.2 (62.2) 12.7 (4.6)	612.4 (359.6) 143.4 (144.5) 16.9 (7.4)	428.5 (247.9) 47.5 (48.5) 10.7 (5.2)	358.8 (273.3) 60.8 (126.6) 9.9 (5.0)	621.4 (294.1) 83.1 (115.6) 15.3 (6.0)	549.5 (248.9) 76.2 (129.5) 12.6 (8.0)	F = 5.86; *p* = 0.004 F = 4.31; *p* = 0.016 F = 8.21; *p* = 0.001

*SD* standard deviation (or standard error where^a^), *MD* mean difference, *OR* odds ratio, *F* F statistic from ANOVA

^†^same study but different sample sizes reported

No meta-analysis for due to baseline differences in vitamin C intake (McAleese, [16]/Christian, [10]) and lack of accounting for possible clustering effects (McAleese, [16])

#### Food preferences

Thirteen studies reported children’s food preferences, including willingness to taste and taste ratings for fruits and vegetables [15, 25, 26, 29–38] (Table 9). Eight of the studies reported statistically significant intervention effects showing increased preference for fruits and vegetables [15, 30, 32–34, 36–38]. However, these are subjective measures which are highly susceptible to social desirability bias, especially in children who may be eager to please after learning about healthy foods and growing vegetables during school gardening time. We consider food preferences as an early step on the pathway towards behaviour change, but not indicative of behaviour change in itself.

**Table 9:** Results of included quantitative studies: child’s food preferences (including willingness to taste and taste ratings)

First author (year) Sample (*n*)	Outcome measures	Outcomes	Intervention group		Comparison group		Control group		Group x time results (adjusted, if reported)
			Baseline Mean (SD)	Follow-up Mean (SD)	Baseline Mean (SD)	Follow-up Mean (SD)	Baseline Mean (SD)	Follow-up Mean (SD)	
Block (2009) [25] *n* = 770	SAKG child questionnaire (four point scale)	Always willing to try new foods if…			NA	NA			NR
		Never tried it before (%) Cooked it (%) Grown it in the garden (%)	26 32 26	39 51 39			35 39 35	23 34 23	
Gibbs (2013) [26] *n* = 764 (some differences)^a^									
	SAKG parent questionnaire	Child always willing to try new foods (%)	27	33			24	27	
Gatto (2012) [29] *n* = 107	Motivation for Healthy Behaving (17 items)	Preference for fruits Preference for vegetables	NR	Change values only reported	NA	NA	NR	Change values only reported	*p* = 0.9 *p* = 0.06
	Combination of measures (13 items; seven point scale)	Fruit from the garden tastes better than fruit from the store (/7) Vegetables from the garden taste better than vegetables from the store (/7)	4.9 (2.4) 4.4 (2.5)	6.2 (1.4) 5.8 (1.8)			4.8 (2.2) 4.2 (2.3)	4.8 (2.2) 4.3 (2.2)	NS *p* < 0.05
Jaenke (2012) *n* = 127 [15]	Food preference assessment tool	Overall willingness to taste (/6) Overall taste rating (/30) Taste rating carrot (/5) Taste rating pea (/5) Taste rating tomato (/5) Taste rating broccoli (/5) Taste rating capsicum (/5) Taste rating lettuce (/5)	4.54 (1.50) 18.5 (7.4) 3.7 (1.6) 2.9 (1.8) 2.9 (2.3) 2.6 (1.8) 2.4 (2.1) 4.1 (1.5)	Between group mean differences only	4.50 (1.94) 18.1 (9.0) 3.7 (1.6) 2.8 (1.8) 2.4 (2.3) 2.8 (2.0) 3.0 (2.1) 3.7 (1.9)	Between group mean differences only	3.93 (2.04) 15.5 (8.8) 3.5 (1.8) 2.0 (1.9) 2.5 (2.4) 2.1 (2.1) 2.1 (2.2) 3.3 (1.9)	Between group mean differences only	*p* < 0.001 *p* < 0.001 *p* = 0.071 *p* < 0.001 *p* = 0.03 *p* < 0.001 *p* = 0.12 *p* = 0.02
Morgan (2010) [30] *n* = 127 (some differences)^a^									
		Reported in Morgan paper only:							
		Willingness to taste: Lettuce (proportion) Carrot (proportion) Capsicum (proportion) Broccoli (proportion) Tomato (proportion) Pea (proportion)	0.94 0.89 0.60 0.71 0.60 0.69	0.97 0.92 0.74 0.93 0.76 0.77	0.83 0.89 0.77 0.74 0.56 0.74	0.85 0.88 0.64 0.61 0.48 0.76	0.77 0.82 0.51 0.58 0.60 0.63	0.61 0.70 0.35 0.36 0.40 0.41	0.24 0.14 0.04 0.01 <0.001 0.02
		Would you eat this food as a snack?							
		Lettuce (proportion) Carrot (proportion) Capsicum (proportion) Broccoli (proportion) Tomato (proportion) Pea (proportion)	0.60 0.67 0.22 0.06 0.46 0.21	0.68 0.60 0.43 0.40 0.48 0.61	0.54 0.64 0.26 0.18 0.48 0.24	0.69 0.60 0.29 0.18 0.32 0.32	0.39 0.63 0.23 0.19 0.42 0.25	0.30 0.61 0.29 0.06 0.34 0.11	0.15 0.89 0.39 <0.001 0.31 0.001
Meinen (2012) [31] *n* = 404	Student survey (three point scale)	Willingness to try fruits and vegetables: If given a new kind of fruit at home (/3) If given a new kind of fruit at school (/3) If given a new kind of vegetable at home (/3) If given a new kind of vegetable at school (/3) Would you choose fruit as a snack? (/3) Would you choose vegetables as a snack? (/3)	2.5 (0.60) 2.2 (0.72) 2.2 (0.70) 2.1 (0.73) 2.4 (0.68) 1.8 (0.74)	2.6 (0.59) 2.3 (0.72) 2.3 (0.70) 2.1 (0.78) 2.5 (0.63) 2.0 (0.73)	NA	NA	2.6 (0.58) 2.3 (0.69) 2.3 (0.69) 2.0 (0.71) 2.5 (0.66) 1.9 (0.78)	2.5 (0.65) 2.2 (0.69) 2.2 (0.71) 2.0 (0.75) 2.5 (0.64) 2.0 (0.75)	NR
	Parent survey (10 items; four point scale)	Like/dislike of fruits and vegetables:							
		Apples (/4) Watermelon (/4) Broccoli (/4) Tomatoes (/4) Spinach (/4) Swiss chard (/4) Zucchinis (/4) Cucumbers (/4) Green beans (/4) Peppers (/4)	3.8 (0.45) 3.7 (0.58) 2.8 (1.05) 2.3 (1.12) 2.2 (0.99) 1.6 (0.82) 2.3 (0.99) 3.0 (0.99) 3.3 (0.89) 2.4 (1.13)	3.7 (0.57) 3.6 (0.68) 2.9 (1.06) 2.5 (1.15) 2.4 (1.14) 2.0 (0.95) 2.4 (0.95) 3.0 (1.05) 3.4 (0.90) 2.6 (1.12)			3.8 (0.46) 3.6 (0.74) 2.9 (1.02) 2.4 (1.13) 2.2 (1.06) 1.7 (0.96) 2.3 (1.11) 2.9 (1.13) 3.3 (0.95) 2.3 (1.17)	3.8 (0.52) 3.6 (0.75) 2.8 (1.07) 2.5 (1.16) 2.1 (1.02) 1.7 (0.78) 2.3 (1.01) 3.1 (1.01) 3.4 (0.85) 2.3 (1.09)	
Morris (2001) [32] *n* = 97	Student questionnaire (six items; five point scale)	Mean total tasting score indicating willingness to taste (/5) Vegetables tasted: spinach, carrots, peas, broccoli, zucchini and red bell pepper.	4.07 (0.31)^a^	4.83 (0.23)^a^	NA	NA	3.90 (0.30)^a^	3.90 (0.29)^a^	*p* < 0.005
Morris (2002) [33] (1) *n* = 213	Vegetable preference survey (six items; five point scale)	Vegetable preference score at post-test:	NR	Post-test:	NR	Post-test:	NR	Post-test:	
		Broccoli (/5) Carrots (/5) Jicama (/5) Snow peas (/5) Spinach (/5) Zucchini (/5)		3.8 (0.1)^a^ 4.7 (0.1)^a^ 3.9 (0.2)^a^ 3.8 (0.2)^a^ 3.0 (0.2)^a^ 4.0 (0.2)^a^		3.8 (0.1)^a^ 4.7 (0.1)^a^ 3.8 (0.2)^a^ 3.1 (0.2)^a^ 3.2 (0.2)^a^ 3.2 (0.1)^a^		3.2 (0.2)^a^ 4.4 (0.1)^a^ 3.6 (0.2)^a^ 2.9 (0.2)^a^ 3.1 (0.2)^a^ 3.1 (0.2)^a^	F = 4.840; *p* < 0.01 F = 5.768; *p* < 0.005 NR F = 7.657; *p* < 0.005 NR F = 10.012; *p* < 0.0005
		Vegetable preference score at 6 m follow up:		Follow up:		Follow up:		Follow up:	
		Broccoli (/5) Carrots (/5) Jicama (/5) Snow peas (/5) Spinach (/5) Zucchini (/5)		4.0 (0.1)^a^ 4.6 (0.1)^a^ 3.8 (0.2)^a^ 3.7 (0.2)^a^ 3.4 (0.1)^a^ 4.0 (0.1)^a^		3.7 (0.1)^a^ 4.7 (0.1)^a^ 3.4 (0.2)^a^ 3.0 (0.2)^a^ 3.2 (0.1)^a^ 3.4 (0.1)^a^		3.5 (0.2)^a^ 4.4 (0.1)^a^ 3.2 (0.2)^a^ 3.0 (0.2)^a^ 3.3 (0.2)^a^ 3.2 (0.2)^a^	NR NR NR NR NR NR
Morris (2002) [34] (2) *n* = 215	Vegetable preference survey (six items; yes/no/don’t know)	Vegetable preferences at post-test: Do you eat this food at home? (/6) Would you ask your family to buy this food? (/6) Would you eat this food as a snack? (/6)	NR	Post-test: 3.3 (0.1)^a^ 2.9 (0.2)^a^ 2.4 (0.2)^a^	NR	Post-test: 3.1 (0.1)^a^ 2.6 (0.2)^a^ 2.2 (0.2)^a^	NR	Post-test: 2.7 (0.2)^a^ 1.9 (0.2)^a^ 1.6 (0.2)^a^	F = 4.165; *p* < 0.05 F = 7.181; *p* < 0.005 F = 5.239; *p* < 0.01
		Vegetable preferences at 6 m follow up:		Follow up:		Follow up:		Follow up:	
		Do you eat this food at home? (/6) Would you ask your family to buy this food? (/6) Would you eat this food as a snack? (/6)		3.2 (0.1)^a^ 2.6 (0.2)^a^ 2.4 (0.2)^a^		3.1 (0.2)^a^ 2.5 (0.2)^a^ 1.9 (0.2)^a^		2.8 (0.2)^a^ 2.4 (0.2)^a^ 1.5 (0.2)^a^	NR NR F = 6.152; *p* < 0.005
O’Brien (2006) [35] *n* = 38	FV preference assessment (four point scale)	Total fruit preference (/8) Total vegetable preference (/16) Fruits and vegetables tasted unknown	7.18 (0.31)^a^ 10.94 (0.92)^a^	7.06 (0.34)^a^ 11.24 (0.92)^a^	NA	NA	6.05 (0.33)^a^ 8.81 (0.91)^a^	6.05 (0.33)^a^ 9.05 (0.97)^a^	NR
Parmer (2009) [37] *n* = 115	FV preference questionnaire (six items; five point scale)	Willingness to taste (/6) Ratings of tasted fruits and vegetables (/5) Fruits and vegetables tasted: carrots, broccoli, spinach, zucchini, cabbage and blueberries.	4.82 (1.6) 3.45 (0.9)	5.50 (1.0) 4.38 (0.5)	5.11 (1.1) 3.85 (0.8)	5.33 (1.2) 4.15 (0.6)	3.84 (2.1) 3.99 (0.7)	4.23 (2.0) 3.82 (0.5)	F = 0.878; *p* = 0.42 F = 14.45; *p* < 0.001
Parmer (2007) [36] *n* = 115									
	FV preference survey (15 items; three point scale)	Fruit preference (/3) Vegetable preference (/3)	2.59 (0.4) 2.08 (0.5)	2.60 (0.3) 2.03 (0.5)	2.70 (0.3) 2.20 (0.6)	2.73 (0.3) 2.14 (0.6)	2.59 (0.4) 2.10 (0.5)	2.57 (0.3) 1.98 (0.5)	NR NR
Ratcliffe (2011) [38] *n* = 320	Taste test (five items; five point scale)	Willingness to taste vegetables (/5) Preference for vegetables (/5) Vegetables tasted: carrots, string beans, snow peas, broccoli and Swiss chard.	NR	Change values only reported	NA	NA	NR	Change values only reported	0.286 0.279
	Garden Vegetables Frequency Questionnaire (22 items plus two added)	Preference for vegetables:							
		all (24 items) grown in school garden (11 items) not grown in school garden (13 items)							0.029 0.017 0.23
		Willingness to taste vegetables:							
		all (24 items) grown in school garden (11 items) not grown in school garden (13 items)							<0.001 <0.001 0.025

*F* fruits, *V* vegetables, *FV* fruits and vegetables, *SD* standard deviation (or standard error where^a^); *OR* odds ratio, *F* F statistic from ANOVA

^a^see results text for explanation of how differences in duplicate data reporting were handled

No meta-analysis due to heterogeneity of outcome measures

#### Knowledge and attitudes towards food

Ten studies reported children’s knowledge and attitudes towards food [10, 30–38] (Table 10). Seven of the studies reported statistically significant intervention effects [10, 30, 33, 34, 36–38]. For the most part these were positive effects, showing improved knowledge or attitudes towards food in the intervention groups. Interestingly, one cluster RCT (Trial 1) in the UK found that students who participated in expert-led gardening activities (intervention group) for 18 months were less likely to have positive attitudes towards fruits and vegetables, compared with students who participated in teacher-led gardening activities (comparison group), suggesting that teacher-led activities might be more effective [10]. However, this study had mixed results with other outcome measures showing no statistical difference between teacher and expert-led gardening. As stated above for children’s food preferences, these measures of knowledge and attitudes are susceptible to social desirability bias and reflect possible behaviour change mechanisms rather than actual behaviour change.

**Table 10:** Results of included quantitative studies: child’s knowledge and attitudes towards food (including self-efficacy)

First author (year) Sample (*n*)	Outcome measures	Outcomes (data are means and SD unless otherwise stated)	Intervention group		Comparison group		Control group		Group x time results (adjusted, if reported)
			Baseline Mean (SD)	Follow-up Mean (SD)	Baseline Mean (SD)	Follow-up Mean (SD)	Baseline Mean (SD)	Follow-up Mean (SD)	
Christian (2014) [10] (2) [9] Trial 1; *n* = 1138	Child questionnaire: FV knowledge Attitudes towards FV	% of children who agreed: I enjoy eating fruit I like trying new fruits I try to eat lots of fruit I enjoy eating vegetables I like trying new vegetables I try to eat lots of vegetables Eating FV every day keeps me healthy	% agreed 94.5 78.0 83.0 65.6 58.9 64.6 93.5	% agreed 91.8 76.3 81.3 64.7 58.0 70.9 94.1	% agreed 96.4 83.3 86.7 66.9 61.0 66.7 94.1	% agreed 96.2 86.6 90.1 65.9 60.0 69.6 97.2	NA	NA	Odds ratio (95 % CI) OR = 0.4 (0.1 to 1.0) OR = 0.5 (0.2 to 0.9) OR = 0.4 (0.2 to 0.9) OR = 1.1 (0.6 to 1.9) OR = 1.0 (0.7 to 1.5) OR = 1.1 (0.7 to 1.7) OR = 0.6 (0.2 to 1.6)
		There is usually lots of FV to eat at home	89.2	89.8	87.6	94.1			OR = 0.4 (0.2 to 0.9)
		I'm good at preparing FV My family encourages me to eat FV % who knew that 5 FV per day are needed to stay healthy	71.8 87.1 76.2	74.7 90.7 79.0	81.3 88.3 72.7	83.6 93.7 79.0			OR = 0.6 (0.3 to 1.1) OR = 0.7 (0.3 to 1.5) OR = 0.9 (0.4 to 1.6)
		% who had tasted their own FV at follow-up	62.3	62.1	52.4	67.8			OR = 0.8 (0.5 to 1.4)
Christian (2014) [10] Trial 2; *n* = 1391	Child questionnaire: FV knowledge Attitudes towards FV	% of children who agreed: I enjoy eating fruit (% who agreed) I like trying new fruits I try to eat lots of fruit I enjoy eating vegetables I like trying new vegetables I try to eat lots of vegetables Eating FV every day keeps me healthy	% agreed 96.7 86.0 87.2 68.8 62.8 72.8 94.9	% agreed 97.6 84.0 88.2 69.5 59.5 75.5 97.0	NA	NA	% agreed 96.8 84.5 82.7 64.2 60.5 66.7 96.2	% agreed 97.0 80.4 85.8 61.7 56.9 68.6 96.4	Odds ratio (95 % CI) OR = 1.1 (0.4 to 2.9) OR = 1.2 (0.7 to 1.9) OR = 1.0 (0.6 to 1.6) OR = 1.2 (0.9 to 1.6) OR = 0.9 (0.7 to 1.2) OR = 1.2 (0.8 to 1.8) OR = 1.2 (0.5 to 2.8)
		There is usually lots of FV to eat at home	89.6	92.8			88.9	89.5	OR = 1.5 (0.9 to 2.5)
		I'm good at preparing FV My family encourages me to eat FV % who knew that 5 FV per day are needed to stay healthy % who had tasted their own FV at follow-up	79.3 89.9 73.6 60.1	78.1 92.8 79.1 66.4			77.9 87.7 67.3 56.0	79.3 91.9 67.5 58.1	OR = 0.8 (0.6 to 1.1) OR = 0.9 (0.5 to 1.6) OR = 1.7 (1.1 to 2.5) OR = 1.4 (0.8 to 2.4)
Meinen (2012) [31] *n* = 404	Knowledge of fruits and vegetables	Knowledge of recommended daily servings of FV (%)	33	35			36	42	NR
Morgan (2010) [30] *n* = 127	Gimme 5 questionnaire (eight multiple choice questions)	FV knowledge (/8)	5.4 (1.4)	Between group mean differences only	5.1 (1.3)	Between group mean differences only	6.1 (1.8)	Between group mean differences only	*p* = 0.02^†^
	Food preference assessment tool	Ability to identify vegetables (/1)	0.9 (0.1)		0.9 (0.1)		0.9 (0.1)		*p* < 0.001^†^
Morris (2001) [32] *n* = 97	Food identification questionnaire (food groups/recommendations)	Nutrition knowledge score (/5)	1.9 (0.2)^a^	2.5 (0.2)^a^	NA	NA	2.4 (0.2)^a^	2.5 (0.2)^a^	NR
Morris (2002) (1) [33] *n* = 213	Nutrition knowledge questionnaire (30 multiple choice questions)	Nutrition knowledge score at post-test (/30):	NR	20.8 (0.4)^a^	NR	20.5 (0.4)^a^	NR	17.1 (0.4)^a^	F = 24.238, *p* < 0.0005
		Nutrition knowledge score at 6 m follow up (/30):		20.8 (0.4)^a^		21.2 (0.4)^a^		18 (0.4)^a^	F = 18.270, *p* < 0.0005
Morris (2002) (2) [34] *n* = 215	Vegetable preference survey (six items)	Ability to correctly name vegetables at post-test (/6)	NR	3.3 (0.1)^a^	NR	3.0 (0.1)^a^	NR	2.6 (0.1)^a^	F = 9.795, *p* < 0.0005
		Ability to correctly name vegetables at follow up (/6)		3.2 (0.1)^a^		2.9 (0.1)^a^		2.8 (0.1)^a^	NR
O’Brien (2006) [35] *n* = 38	Nutrition knowledge questionnaire (derived from Family Nutrition Program evaluations)	Nutrition knowledge (/10)	7.53 (0.34)^a^	7.18 (0.30)^a^	NR	NR	7.05 (0.29)^a^	7.38 (0.33)^a^	NR
	Self-efficacy instrument (Domel et al. 1996)	FV consumption self-efficacy (/10)	8.94 (0.29)^a^	9.06 (0.26)^a^			8.33 (0.33)^a^	8.67 (0.25)^a^	
	Outcome expectations questionnaire (Domel et al. 1995)	FV consumption expectations (/6)	5.76 (0.18)^a^	5.24 (0.27)^a^			5.29 (0.24)^a^	5.52 (0.16)^a^	
Parmer (2009) [37] *n* = 115	Fruit and vegetable survey (adapted Struempler & Raby)	Food groups Nutrient-food associations Nutrient-job associations FV identification	3.69 (1.8) 1.46 (1.1) 1.25 (1.0) 3.14 (0.7)	5.20 (1.2) 3.56 (1.6) 2.97 (1.9) 4.89 (0.9)	4.08 (1.7) 1.67 (1.5) 1.27 (1.3) 3.03 (0.6)	4.75 (1.9) 3.70 (1.8) 2.64 (1.6) 3.44 (0.8)	4.03 (1.8) 1.82 (1.4) 1.71 (1.2) 2.88 (0.9)	4.46 (1.3) 1.92 (1.3) 1.46 (1.0) 2.96 (1.0)	NS F(2,122) = 11.84; *p* < 0.001 F(2,122) = 12.05; *p* < 0.001 F(2,78) = 28.08; *p* < 0.001
Parmer (2007) [36] *n* = 115									
Ratcliffe (2011) [38] *n* = 320	Taste test	Ability to identify vegetables	NR	Change values only reported	NA	NA	NR	Change values only reported	*p* = 0.002

*FV* = fruits and vegetables, *SD* standard deviation (or standard error where^a^); *OR* odds ratio

^†^Note: the p values reported for these outcomes relate to subgroup analysis (*n* = 109) of students with lower baseline scores (Morgan et al. 2010)

No meta-analysis due to heterogeneity of outcomes and different comparison groups (Christian, [10] Trials 1 and 2)

#### Physical health and activity

Two studies reported physical health measures, both including waist circumference, body mass index (BMI), and systolic and diastolic blood pressure [22, 28] (Table 11). The only statistically significant difference was reported in a non-randomised controlled study for diastolic blood pressure, which lowered more in the intervention group (cooking, nutrition and gardening) compared to the control group [28]. However, all of the blood pressure readings in this study were within the normal range for school children (systolic: 85–120; diastolic: 50–80) so this finding may not be clinically relevant as an improvement in physical health. Another cluster RCT (elementary school based) reported physical activity measures derived from both a questionnaire and accelerometry [23] (Table 11). While children who participated in school gardening (intervention group) reported being ‘usually’ less sedentary (*p* = 0.001) and spent more time engaged in ‘moderate’ physical activity (*p* = 0.010) compared to the control group, there was no increase in ‘light’ physical activity or reduction in sedentary behaviour when measured objectively using accelerometers. Note however that the accelerometer analysis was based on a selected subsample of students.

**Table 11:** Results of included quantitative studies: child’s physical health and activity

First author (year) Sample (*n*)	Outcome measures	Outcomes	Intervention group		Comparison group		Control group		Group x time results (adjusted, if reported)
			Baseline Mean (SD)	Follow-up Mean (SD)	Baseline Mean (SD)	Follow-up Mean (SD)	Baseline Mean (SD)	Follow-up Mean (SD)	
Cotter (2013) [22] *n* = 155	Standard clinical measures	Waist circumference (cm) BMI (kg/m^2^) Systolic blood pressure (mmHg) Diastolic blood pressure (mmHg) Urinary sodium (mmol/24 h)	67.8 (8.2) 19.0 (2.7) 117.4 (9.9) 66.9 (8.0) 126.6 (40.5)	68.6 (7.6) 19.3 (2.8) 113.9 (9.6) 66.2 (8.5) 108.2 (37.3)	68.1 (9.0) 19.0 (3.2) 115.1 (14.8) 65.4 (8.2) 138.4 (50.7)	69.9 (8.9) 19.0 (3.1) 111.3 (11.6) 64.8 (7.4) 128.2 (50.9)	69.5 (8.6) 19.1 (3.2) 122.1 (14.1) 73.5 (9.6) 131.3 (34.9)	71.5 (8.1) 19.1 (3.1) 113.9 (9.9) 67.0 (7.4) 125.3 (50.6)	NR
Davis (2011) [28] *n* = 104	Standard clinical measures	BMI (kg/m^2^) Waist circumference (cm) Total fat (%) Systolic blood pressure (mmHg) Diastolic blood pressure (mmHg)	20.4 (4.2) 73.9 (13.3) 28.2 (12.6) 105.9 (8.20) 59.6 (8.4)	20.4 (4.0) 74.9 (13.6) 26.8 (12.4) 101.9 (10.4) 56.5 (5.6)	NA	NA	21.8 (5.1) 75.7 (13.2) 29.0 (9.8) 108.9 (8.9) 60.8 (8.0)	22.0 (5.2) 77.3 (13.9) 27.6 (10.3) 104.5 (9.8) 58.7 (6.2)	*p* = 0.14 *p* = 0.67 *p* = 0.59 *p* = 0.53 *p* = 0.04
Wells (2014) [23]	Physical activity	GEMS Activity Questionnaire:							Mean difference:
*n* = 227 (or *n* = 124 for accelerometry data)		Activity - yesterday Activity - usually Sedentary - yesterday Sedentary - usually	2.91 (0.19) 3.78 (0.18) 0.63 (0.04) 0.78 (0.05)	2.48 (0.20) 3.43 (0.19) 0.51 (0.04) 0.68 (0.05)	NA	NA	2.74 (0.17) 3.61 (0.16) 0.57 (0.04) 0.68 (0.04)	2.51 (0.19) 3.63 (0.18) 0.54 (0.04) 0.77 (0.05)	−0.20; *p* = 0.312 −0.37; *p* = 0.083 −0.09; *p* = 0.064 −0.19; *p* = 0.001
		Accelerometry:							Mean difference:
		Sedentary (%) Light PA (%) Moderate PA (%) Vigorous PA (%) MVPA (%)	55.23 (1.71) 34.62 (1.00) 5.17 (0.54) 5.01 (0.58) 10.14 (1.03)	55.00 (1.73) 33.17 (1.02) 5.62 (0.54) 6.24 (0.59) 11.82 (1.04)			54.75 (1.59) 35.09 (0.92) 5.41 (0.50) 4.99 (0.54) 10.35 (0.95)	56.11 (1.60) 33.07 (0.93) 5.28 (0.50) 5.78 (0.54) 11.03 (0.95)	−1.59; *p* = 0.144 +0.57; *p* = 0.492 +0.58; *p* = 0.010 +0.44; *p* = 0.213 +1.00; *p* = 0.044

*SD*standard deviation (or standard error where^a^)

No meta-analysis due to lack of adjustment for possible clustering effects

#### Well-being

Four studies reported children’s well-being using a variety of measures, only some of which used valid and reliable scales, including quality of life, life skills and interpersonal relationships [17, 24, 30, 39] (Table 12). Two of the four studies did not find a significant difference between intervention and control groups using their selected measures at follow up [24, 30]. The other two studies did not report their child wellbeing outcomes adequately [17, 39].

**Table 12:** Results of included quantitative studies: child’s well-being (including social skills)

First author (year) Sample (*n*)	Outcome measures	Outcomes	Intervention group		Comparison group		Control group		Group x time results (adjusted, if reported)
			Baseline Mean (SD)	Follow-up Mean (SD)	Baseline Mean (SD)	Follow-up Mean (SD)	Baseline Mean (SD)	Follow-up Mean (SD)	
Block (2012) [24] *n* = 764	KIDSCREEN-10	Child quality of life score	48.9 (8.4)	50.3 (8.1)	NA	NA	48.2 (7.9)	49.1 (7.3)	Adjusted statistic = 1.23 (0.7); *p* = 0.09
	Teacher questionnaire	Teacher strongly agrees that:							
		Student social behaviour is good (%)	41.9	48.9			41.9	53.8	*p* = 0.2
		Students cooperate well with other students (%)	48.8	57.8			48.4	65.4	Adjusted statistic = 0.51; *p* = 0.3
Morgan (2010) [30] *n* = 127	Quality of school life instrument (40 items)	Quality of school life	3.2 (0.2)	Between group mean differences only	3.2 (0.3)	Between group mean differences only	3.0 (0.4)	Between group mean differences only	*p* = 0.98
Robinson (2005) [39] *n* = 281	Youth Life Skills Inventory (32 questions; three point scale)	Overall life skills score (/96) Working with groups Self-understanding Leadership Decision making Communication Volunteerism	83.02 (7.95) 13.33 (1.74) 16.78 (1.96) 12.62 (2.05) 13.71 (1.64) 10.59 (1.55) 16.57 (1.77)	84.51 (7.81) 14.09 (1.41) 18.02 (1.76) 12.63 (1.85) 13.72 (1.44) 10.42 (1.46) 16.23 (2.08)	NA	NA	85.8 (6.14) NR NR NR NR NR NR	86.49 (6.19) NR NR NR NR NR NR	NR
Waliczek (2001) [17] *n* = 589	Self-Report of Personality Scale for children and adolescents	Interpersonal relationships	Means by age and gender only	Means by age and gender only	NA	NA	Means by age and gender only	Means by age and gender only	NR

*SD* standard deviation

No meta-analysis due to heterogeneity of outcome measures

Overall, quantitative evidence for the impacts of school gardens is limited with some suggestion of improvements in knowledge, attitudes and preferences towards fruit and vegetables, but little objective evidence of changes in eating habits or physical health benefits.

### Qualitative evidence for the health and well-being impacts of school gardening

Qualitative evidence was synthesised narratively and is described below in relation to the health, wellbeing and educational impacts described by children, teachers and parents, and those factors thought to be associated with the success and sustainability of school gardens. Tables 13 and 14 show the subthemes which make up each of these broad themes and which studies contributed to each.

**Table 13:** Contribution of individual qualitative studies to descriptive themes: Health and well-being impacts of school gardening

First author (year)	Quality	Health impacts				Well-being impacts						
		Food/nutrition knowledge	Attitudes towards food	Healthier eating habits	Physical activity	Enjoyment	Achievement, satisfaction, pride	Confidence, self-esteem, ownership, responsibility	Relaxation, stress release	Express/manage emotions	Building relationships, belonging	Cultural awareness, cohesion
Ahmed (2011) [40]	Moderate		✓	✓	✓		✓					✓
Alexander (1995) [41]	Moderate					✓	✓	✓		✓	✓	
Anderson (2011) [42]	Weak											
Block (2009, 2012) [24;25] Gibbs (2013) [26] Townsend (2014) [43]	Strong	✓	✓	✓		✓	✓	✓	✓	✓	✓	✓
Bowker (2007) [44]	Moderate			✓		✓	✓	✓			✓	
Chawla (2014) [11]	Strong					✓	✓	✓	✓	✓	✓	
Chiumento (2012) [12]	Weak					✓	✓	✓	✓		✓	✓
Cutter-Mackenzie (2009) [45]	Moderate			✓		✓			✓			✓
Hazzard (2011) [46]	Weak											
Henryks (2011) [47]	Moderate	✓	✓	✓		✓	✓	✓			✓	
Lakin (2008) [48]	Weak	✓	✓	✓		✓	✓	✓				
Miller (2007) [49]	Weak	✓					✓	✓		✓	✓	
Ming Wei (2012) [13]	Moderate			✓	✓	✓		✓	✓	✓	✓	
Passy (2010) [50]	Moderate	✓	✓	✓	✓	✓	✓	✓		✓	✓	✓
Somerset (2005) [51]	Weak	✓	✓		✓	✓	✓	✓		✓	✓	
Viola (2006) [52]	Moderate	✓		✓	✓	✓		✓			✓

**Table 14:** Contribution of individual qualitative studies to descriptive themes: Educational impacts & factors influencing the success of school gardens

First author (year)	Educational impacts				Factors influencing success and sustainability					
	Academic improvements	Student engagement, motivation	Environmental awareness	Development for staff/volunteers	Experiential learning style, curriculum integration	Supportive environment, inclusive, equal	Cultural relevance	Support from staff, volunteers community	Pressure on staff, volunteers, timetable	Fundraising, resources
Ahmed (2011) [40]	✓				✓		✓			
Alexander (1995) [41]		✓	✓					✓	✓	
Anderson (2011) [42]										
Block (2009, 2012) [24;25] Gibbs (2013) [26] Townsend (2014) [43]	✓	✓	✓	✓	✓	✓	✓	✓	✓	✓
Bowker (2007) [44]	✓		✓		✓					
Chawla (2014) [11]	✓		✓							
Chiumento (2012) [12]		✓				✓	✓			
Cutter-Mackenzie (2009) [45]			✓		✓		✓	✓		
Hazzard (2011) [46]					✓			✓	✓	✓
Henryks (2011) [47]				✓		✓		✓		
Lakin (2008) [48]			✓		✓			✓		
Miller (2007) [49]	✓		✓					✓		
Ming Wei (2012) [13]	✓	✓				✓		✓		✓
Passy (2010) [50]	✓	✓	✓	✓	✓	✓		✓	✓	✓
Somerset (2005) [51]	✓				✓			✓		✓
Viola (2006) [52]					✓		✓	✓		

#### Health impacts

Most studies described perceived nutritional benefits for children involved in school gardening, including greater knowledge and awareness, improved attitudes towards food such as willingness to try new foods, and healthier eating habits [13, 25, 40, 44, 45, 47–52] (Table 13).

*When I grow them [vegetables] I feel like I should always try it. And when I’ve grown them I like them better than the shop ones.* – Child, primary school [50].

This quote illustrates how participation in school gardening created a sense of connection to the food grown, which encouraged children to be more adventurous and led to increased preference for vegetables. Staff and volunteers observed these changes in children’s attitudes and behaviour and some, like this administrator, became optimistic about the potential of school gardens to generate major shifts in food culture in the long term.

*We’ve got to start with these kids now, so that when they become the grandparents, they’re modelling correctly for the kids. We’re probably not going to change the values of today’s elderly and today’s parents, but if we begin with the kids we’re going to have a chance over time to change the health and wellness of the population.* – Administrator [40]

These kinds of aspirations may be idealistic, but they contributed to enthusiasm and motivation to support the school gardens, the importance of which will be explored later in this review. The quote also illustrates the perception expressed in three studies [40, 50, 51] that starting at an “early” age (not defined) could be an important factor in the success of school gardening interventions for encouraging the development of a healthy lifestyle.

Other studies described school gardening as an opportunity for physical activity for both children and adults [13, 40, 50–52]. Primary school teachers appreciated the physical aspect of gardening for certain groups of students in particular: “the boys that you want to keep physically busy” and those “children who cannot concentrate in class” [51]. In a rural Hawai’ian elementary school, parents saw it as an opportunity “to teach work ethics, to become physically strong and healthy, and to raise awareness of how other people labour to make our lives better and easier” [13]. School gardens are positioned as part of a social, even moral, education.

#### Well-being impacts

Most qualitative studies reported well-being impacts of school gardening for children and/or adult participants [11–13, 25, 40, 41, 44, 45, 47–52] (Table 13). We further categorised these into personal and social well-being impacts. The personal well-being impacts included enjoyment and feelings of achievement, satisfaction and pride from nurturing the plants, seeing them grow and eventually harvested the crops [11–13, 25, 40, 41, 44, 45, 47–52].

*It makes me feel good inside, all fresh, good… I enjoy touching the soil, the plants. You can feel them…I feel part of them…Yes, it makes me feel that I can care more about things… Being more gentle, caring more, the plants are like people.* – Student, age 17 [11].

These emotions are visceral and again there is the sense of connection to nature, which is very different to the classroom experience and brings different lessons – based on empathy and care – to the children in terms of how they interact with people.

Most studies described how children gained confidence and self-esteem through school gardening [11–13, 25, 41, 44, 47–52]. Developing and maintaining the gardens gave children the opportunity to demonstrate ownership and responsibility [12, 13, 24, 40, 48–50], which may have contributed to these feelings of confidence.

*A child who struggled and had learning disabilities … and just her confidence and her ability to outshine other kids, who have strengths in other areas was just amazing and she was just really comfortable, in her element. She knew exactly what she was doing, she was in control, she was starring while she was organising the other kids. The building of confidence was just amazing.* – Teacher, primary school [25].

This quote echoes those above and suggests particular benefits of mastery and empowerment for children who do not excel in the usual academic setting, such as those with learning or behavioural difficulties. The school gardens allowed them to shine in different ways and to experience success.

In some studies, children and adolescents described school gardens as peaceful places (using words like ‘refuge’ or ‘sanctuary’) where they could slow down and let go of any stresses [11–13, 24, 45]. Students who reported these kinds of benefits included some with mental health disorders like Attention Deficit Hyperactivity Disorder (ADHD) and depression [11], behavioural and emotional difficulties [12] and minority ethnic groups including recent migrants [45]. Teenage gardeners articulated reasons why they found the garden so relaxing [11]. For some, it was about being outdoors and the connection with nature, which gave them a sense of perspective. For others, the contrast of physical work allowed the brain some quiet time for reflection and this enabled them to process stress.

*It's almost like meditation, like my body is present but my mind just kind of drifts off and goes someplace else, and thinks about things…It's brainless tasks most of the time, so it's also like zenful, so you get to listen to things…I think about stuff, so I don't have to go home and think about it right before bed, so instead I can just go to sleep and stuff. I just feel happier in a way, and more at peace.* – Student, age 15 [11].

This is another example of the sensory, visceral nature of gardening activities, which may have stress-reducing or restorative effects similar to those described in Ulrich’s psycho-evolutionary theory [53].

Children experienced positive and negative emotions in the school gardens and participants described how they were able to express themselves and manage their emotions more effectively in that environment [11, 13, 25, 41, 49–51]. For example, following the vandalising of the school garden, a teacher said:

*It really offended them that these students had done this damage to their garden…So then we talk about it and say, well, it made them feel very angry that these children had destroyed part of their garden…it was a positive experience that the children learn that doing what to those children must have been a fun thing to do to go tear up our garden, didn't make us feel good. They were on the receiving end of it and so even though it was a negative experience you can make it a positive one.* – Teacher [41].

In this example, the teacher had her own views about how the negative experience has been turned into a constructive learning experience, but this was not articulated by the children themselves.

The social well-being impacts of school gardening were mainly about building relationships [11–13, 25, 41, 44, 47, 49–52]. Children enjoyed interacting with their friends, teachers, gardening specialists, parents and volunteers – some of whom were people they would not normally come into contact with.

*You have to work together….It’s not about individualism which is promoted in the school structure in some ways, but really communicat[ion], cooperation and ownership of something.* – Garden staff [40].

Gardening was seen as promoting teamwork and cooperation, working together towards common goals, which may help to break down some of the social boundaries and elitism associated with traditional academic structures.

Teachers, parents and volunteers also enjoyed the opportunity to interact with children in the garden setting and described how it improved teacher-student and intergenerational relationships [25, 47, 51]. Volunteers described feeling valued and ‘belonging’ in the school gardens [43, 47]. This gave them a sense of purpose because they felt they were doing something worthwhile – contributing to the children’s education and giving back to the community. In this respect, the personal and social well-being impacts of school gardening are interrelated and this combination of factors creates the motivation for volunteering.

Finally, participants described how school gardening contributed to improved cultural awareness and cohesion [12, 25, 40, 45, 50]. Parents from non-English-speaking backgrounds who might not contribute to other school activities felt comfortable in the garden [24]. In multi-cultural communities, the combination of gardening and cooking activities provided an opportunity for children to learn about each other’s cultures [45]. It was also a successful medium for developing English language skills as children relaxed and engaged in “everyday conversations” [45].

### Qualitative evidence for educational impacts of school gardening

The most common outcomes reported alongside health and well-being impacts were related to potential educational impacts of school gardening [11–13, 25, 40–42, 44, 45, 47–51] (Table 14). As we have included only studies also reporting health and well-being impacts, we can only present evidence for the educational impacts of school gardening from these studies, and so this is not a comprehensive synthesis of the qualitative evidence for the educational impacts of school gardening. Six studies which focussed on educational aspects were excluded during full text screening because they did not match our primary review focus on health and well-being outcomes (see Fig. 1).

Whilst none of the included studies reported academic attainment outcomes (quantitative), two qualitative studies reported children’s beliefs that school gardening was having a positive impact on their school work [11, 44] and these beliefs were echoed by teachers and volunteers in four more studies [13, 25, 50, 51].

*I'm able to complete my homework faster, because I'm in a better place to do other things, because I just spent an hour not worrying about my homework and my grades and my timing for anything, because there's no deadline here.* – High School Student with ADHD, age 17 [11]

This quote provides further evidence that it might be children with special educational needs that stand to benefit the most from school gardening. It also suggests that well-being impacts such as stress reduction may lead to academic impacts for some students, echoing the proposed mechanisms of Attention Restoration Theory, which suggests that contact with nature can restore depleted ability to concentrate [54].

Increased levels of engagement and motivation among children who participated in school gardening was noted in some studies, although it was not always clear if this was referring generally to time spent in the garden, to garden activities intended to promote academic learning, or also to classroom-based learning [12, 13, 24, 41, 50]. In one study, teachers described how they harnessed this potential by tailoring gardening activities to the needs of individual children and creating opportunities for them to demonstrate their skills and knowledge [24]. In our view this has obvious links to the well-being impacts of confidence and self-esteem. However, some children did not work well in the gardens and teachers found them difficult to ‘contain’ in open spaces [25]. Suggested reasons for this were that some tasks were repetitive and the rewards of gardening were not immediate, resulting in loss of engagement [25].

Some children developed a greater awareness of the environment through school gardening [11, 25, 41, 44, 45, 48–50]. This varied from awareness of the immediate garden environment, such as water conservation, seasonality, composting techniques and local wildlife [25, 44] to consideration of global environmental issues, such as the food supply chain (“ground to plate”), sustainability, recycling and the importance of protecting the environment [44, 45, 48]. Gardening was seen as a positive environmental behaviour and reinforced the sense of connection with nature [11, 45, 48].

There were also ‘educational’ impacts for adult staff and volunteers in terms of learning new skills in the school gardens and related activities like cooking [25, 47, 50]. In some schools there were formal opportunities for volunteers to attend short courses and gain certificates, which helped to keep volunteers motivated [43].

### Qualitative evidence for factors influencing the success and sustainability of school gardening programmes

Most qualitative studies discussed one or more aspects of school gardening programmes that had contributed to their success and/or challenges they had to overcome, evidence which will be useful for schools considering, implementing or managing a gardening programme [12, 13, 25, 40, 41, 44–52] (Table 14).

The ‘experiential’ or hands-on learning style was described as an effective way to teach children academic subjects in a more applied and holistic way [25, 40, 44–46, 48, 50–52].

*We’ve done a lot of graphs, a lot of growth measurement. Planted seedlings, measured them and predicted at sixteen weeks, forecasting what size they will be. They are graphed and monitored every fortnight… And we’ve talked about sustainability, compost and everything just ties in…We’ve used maths, perimeter, and volume in the garden…Cubic metres…That would have been a really good one for the [grade] five/sixes, if they had actually bought the soil, found out the costing. A lot of things like that you think of in retrospect…There’s still more scope to have time in the regular curriculum and a more consistent approach, to have more of a strategic approach*. – Teacher [25].

This example from the Stephanie Alexander Kitchen Garden Program illustrates the potential for schools to integrate core curriculum subjects with fun gardening activities, and suggests a strong mechanism underpinning the potential well-being and educational impacts of school gardens. However, not all the interventions studies in this review integrated garden and curriculum activities in this way (Table 4).

Some qualitative studies indicated that some form of cooking or food preparation was integrated with the school gardening intervention, or encouragement to become involved in cooking or food preparation at school or at home, for some or all of their participants (but this was not necessarily a key component of the integration – see Table 4) [13, 24, 42, 45, 47, 48, 50, 52].

*Doin’ the cook-up with Miss…was fun. We put a recipe book together for the tuckshop as well. We did this every week so that the tuckshop would have healthy food*. – Student [52].

Cooking facilitated enjoyment and a sense of achievement. Passy also suggests that the sense of celebration created by sharing cooked garden produce was also important in encouraging students to taste the food [50].

*We have a whole bunch of young adults who know how to go to the shop or the market and pick up some vegetables and make themselves something delicious out of it…imagine uni students nourished on seasonal vegetables instead of two-minute noodles.* – Volunteer [47].

This example, from the Stephanie Alexander Kitchen Garden Program, illustrates how combining experience of gardening and cooking also gave children some of the life skills needed to live healthily [47]. It also supports the earlier quote from Ahmed et al. (2011) which proposes the potential long term impacts of changing attitudes to food through gardening.

School gardens were also described by teachers, parents and volunteers as being supportive and inclusive environments; a ‘level playing field’ where all children could participate equally [12, 13, 24, 47, 50]. This characteristic may help to explain well-being benefits if children felt comfortable to be themselves and participate without any sense of pressure or competitiveness. One study described how achievements in the garden were celebrated in school newsletters or assemblies, which contributed to feelings of pride, confidence and motivation among children who were previously unruly [50].

Considering the socio-demographic and cultural characteristics of the school community in the design of school gardening programmes could ensure that they effectively engage both children and the wider community [12, 25, 40, 45, 52]. This connection with cultural heritage and local foods was particularly important in multi-ethnic or native/indigenous communities.

In eleven studies and across many different types of schools, support from stakeholders – including staff, volunteers and the wider community – was considered one of the most important factors influencing the success and sustainability of school gardens [13, 25, 41, 45–52].

*I bring in a variety of people throughout the year to help with various facets of our garden … Last week we had someone come in and show us how to prune our fruit trees and so they get all different kinds of role models…some people are perhaps more patient, some people are less patient....they realize that not all men are the same, not all women are the same, and they get to see people who are not teachers.* – Teacher.

This quote relates to gardening specialists, but support from volunteers (including parents, grandparents and other members of the community) and local organisations/businesses was also valued by teachers for similar reasons – increasing capacity, diversity of skills, materials and resources. However, some schools experienced difficulty recruiting enough volunteers [25, 46, 50].

*The four of us especially have all realized they need some support for this kitchen garden program, being that our funding runs out at the end of this year. So we ran this bloody fair and that was six months of my life and that’s what I gave up to ensure that my children still have this program in their school.* – Parent volunteer [43].

This is clearly an example of overdependence on volunteers, which became a source of resentment and threatened the sustainability of the garden. This suggests that schools need to consider the balance between making use of volunteers and keeping them motivated. The same applies to teachers (and other school staff) and two studies highlighted concerns about increased pressure on workloads and fitting school gardening into an already overcrowded timetable [25, 50]. One innovative way of motivating teachers was to offer continuing professional development (CPD) opportunities, benchmarks to work towards and cash prizes [50]. A study of the California Instructional School Garden Program found that when schools formed a garden committee (including administrators, teachers, parent/community volunteers and garden coordinators) it helped to define roles, share responsibilities and reduce the risk of overburdening any one person [46].

Finally, some schools experienced financial challenges, such as securing ongoing funding and resources for the school gardens [13, 25, 46, 50, 51]. Schools had found various solutions included fundraising events, donations from local businesses and grant applications [25].

*There is a lot of sharing that goes on within the gardening community, and I think it’s important to reach out beyond the school gardens and contact people in community gardens and local gardening clubs. I have also contacted all of the retailers in this area – all the big box stores, the local nursery stores if they have damaged goods – if they have goods that are unsalable in any way, if they’re just old seeds, I’ll take them.* – Member of staff [46].

This example demonstrates the importance of developing links with the wider community to increase the visibility and sustainability of the school garden.

## Discussion

In this mixed methods review, we have systematically and transparently identified, selected, appraised and synthesised the best available evidence on the health and well-being impacts of school gardens. We have used the highest quality international evidence available, although much of this, particularly the quantitative evidence, was judged to be weak in our quality appraisal (Tables 5 and 6).

We found some quantitative evidence for nutritional impacts of school gardening, such as increased preference for, and consumption of, fruits and vegetables. However, many of the included studies relied upon self-reported outcomes, likely to be affected by social desirability bias, especially in children in school settings. It is notoriously difficult to measure food consumption accurately, with different measures having different challenges – the CADET tool, for example, has been found to overestimate fruit intake and underestimate vegetable intake [55]. It was not possible to conduct meta-analyses due to study design and data limitations. Measurement scales, or methods of applying the same measures, were too heterogeneous to allow outcomes to be pooled. In addition, interventions ranged in length from 10 weeks to 18 months.

We also found substantial qualitative evidence on a wide range of health and well-being impacts but these were rarely supported by the quantitative evidence, either because these outcomes were not measured, or because few studies identified significant impacts. It is not clear why these perceptions in the qualitative evidence synthesis are supported by few findings in the quantitative evidence base; whether this should be treated as evidence of no effect or whether, especially due to limitations in the quantitative study designs, it should be seen as no evidence of effect. It should also be noted that only three studies used mixed methods to evaluate impacts, and the quantitative and qualitative evidence comes largely from different school gardening interventions.

Our qualitative synthesis provides contextual information about which aspects of school gardening may be important, how health and well-being impacts may be related to educational impacts, and what factors are important for the success and sustainability of school gardening programmes. This qualitative evidence provides plausible suggestions for *how* school gardens could lead to health and well-being improvements which may help to influence better study design and the elements of school gardens that have the potential to be beneficial.

**Fig. 3:**
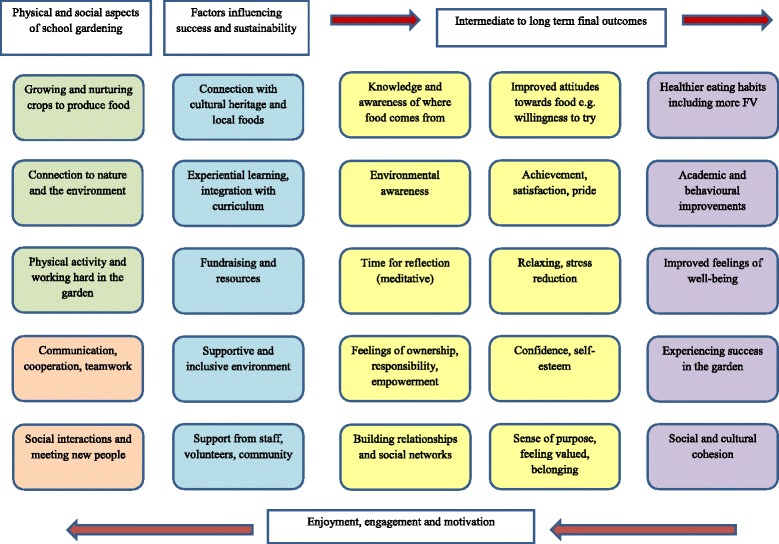
Conceptual model showing the potential health and well-being impacts of school gardening

Based on the qualitative synthesis, we have developed a conceptual model (Fig. 3) to visually represent some of the possible mechanisms and pathways through which gardening could lead to health and well-being impacts. These are our interpretations of the evidence we have synthesised and, to some extent, the model has been left open to further interpretations, without the use of lines joining up specific pathways. It should be read from left to right to consider how the physical and social aspects of school gardening, coupled with factors influencing success and sustainability, might lead to health and well-being (and other) outcomes. The bottom arrow suggests a feedback loop mediated by feelings of enjoyment, engagement and motivation. This model builds on the ‘social-ecological conceptual model’ presented in a previous review, which depicted potential short-term (proximal) and long-term (distal) effects of school gardens and the interconnections between individual, family, school and community-level effects [3]. Our model also suggests the potential for broader intermediate and long term impacts, although we have focused on more immediate, individual-level health and wellbeing effects as determined by our original review questions. Such long term effects are supported in the broader literature, particularly those suggested that understanding and appreciation of the natural world in childhood may lead to environmental responsibility in adulthood as well as support broader perceptions of wellbeing and quality of life [56, 57].

Although much of the evidence from individual qualitative studies is context specific, we have observed several cross-cutting themes that we believe to be transferable between studies. Firstly, school gardening can be integrated with the wider curriculum to maximise opportunities for learning: from nutrition education, to practical growing and food preparation skills, to core curriculum subjects taught in fun ways. To achieve this it is important for teachers to be involved in developing and delivering school gardening activities, with support from other stakeholders in the school and community. Secondly, school gardens appear to have particular benefits for children who have complex needs (behavioural, emotional, or educational) and do not thrive in an academic environment. The evidence suggests that these children may be able to express themselves better in the garden, leading to feelings of calmness, self-esteem and success. Gardening may therefore be described as physical, social and visceral; distinct and complementary to the individual and cerebral nature of classroom education. Thirdly, we noticed a two-way flow whereby the perceived benefits associated with school gardening meant that children were motivated to continue gardening and adults (teachers, parents and volunteers) were motivated to continue to support the school gardening programmes. This feedback loop contributes to the ongoing success and sustainability of school gardening programmes, as indicated in our conceptual model (Fig. 3).

### Strengths and limitations of the review

By combining quantitative and qualitative synthesis methods, this review has highlighted the divergence between these research methods and the need for greater synergy. The qualitative research suggests that health and well-being impacts may be felt by those children who struggle in a classroom setting, but quantitative studies did not examine this subgroup. It is possible that average population outcomes obscure impact among these children, or that the outcomes are less relevant to them. Although improvements in eating habits and physical activity were reported in the qualitative research, these were poorly supported by the quantitative studies. Again, it is unclear whether this is due to lack of effect, or deficiencies in the study designs. The qualitative research suggests holistic effects that may be difficult to quantify, as well as suggesting that impacts may be felt in the medium to long term, whereas included studies report only short term follow up.

Whilst the qualitative studies provided the greatest insights, we recognise that by combining multiple studies set in different contexts, some of the meaning and depth of findings from individual studies will have been lost. Whilst we added our own interpretations to those of the authors, we were limited by the original study designs and implicit biases. For example, the primary studies did not consider the perspectives of children who did not participate in school gardening and reasons for this.

School gardens can be seen as operating in line with the WHO Health Promoting School’s Framework which aims to take a holistic approach to health promotion in schools [58]. Our focus was health and well-being impacts and we did not include studies that focused only on educational impacts of school gardens. Future reviews could consider this broader remit which may be important for school policy makers.

We had hoped to comment on whether there were different impacts of school gardening interventions on health and wellbeing for different age groups. However, the majority of studies focus on younger children, in pre-school [21] or primary/elementary school [9, 15–17, 24–31, 33, 35, 37, 39] with only a small number of participants of middle school [22, 38] or junior high age [17] (Table 2). We therefore conclude that there is insufficient evidence to answer this question.

We restricted this review to OECD countries because developing countries have very different baseline health (and nutrition) characteristics and needs. Most of the evidence comes from the UK, Australia and the USA and it is unclear how transferable the findings are beyond these locations.

### Implications and recommendations for future research

The quality appraisal of both quantitative and qualitative studies included in this review highlighted weaknesses in study design and reporting, despite using strict criteria to exclude the weakest study designs. We would recommend that future studies apply the quality criteria used in systematic reviews at the design stage to improve the robustness of the findings and facilitate meta-analysis. More convincing quantitative evidence is needed to promote school gardening programmes as public health interventions. Greater use of objective measures would provide more robust evidence and consistency in measures used across studies would allow meta-analysis in future reviews.

Our findings have some resonance with theories identified in a recent systematic review of how the school environment impacts on student health [59]. For example, the ‘theory of human functioning and school organisation’ suggests that the ways in which schools implement formal and informal modes of teaching, and develop relationships between staff and students, influence students’ commitment and engagement to learning [60]. The ‘social development model’ suggests that pro-social activities can increase students’ commitment to school [61]. Activities that support social and emotional learning have the potential to reduce stress and improve behaviour, both of which may ultimately improve school performance [62]. Although gardening was not one of the mainstream activities tested in the meta-analysis by Durlak et al., it has the potential to be. Considering in more detail *how* school gardens are anticipated to impact on the school experience, student health and well-being and school outcomes, including through the development of logic models or theories of change, garden programmes’ impact could be enhanced.

A set of theories in the broader literature suggest that school gardening may have longer term impacts than those addressed in the studies included in this review. Positive and repeated contact with elements of the natural world in childhood has been suggested to relate to pro-environmental behaviours and beliefs in adulthood [57]. Pro-environmental behaviours and ‘connectedness to nature’ have been found to be related to dimension of wellbeing [63]. Furthermore the type and frequency of childhood exposure to natural environments is thought to influence adult use of such spaces [64]. There is a growing body of evidence which has shown robust associations between use of natural environments (for leisure, physical activity and so on) and a range of positive health outcomes [65]. There is therefore interest in identifying ways in which children can be provided with regular and meaningful opportunities to experience the natural environment.

Future studies on school gardening could usefully make use of theory-led methods, such as realist synthesis or evaluation [66, 67], to develop evidence-based causal explanations of how and why school gardens work, for which groups of students, in which types of schools.

## Conclusions

Despite their popularity, there is currently limited quantitative evidence that school gardens can have health and well-being benefits for students, and the evidence that does exist is based on self-reported outcome measures. The qualitative evidence suggests that participants in school gardening programmes (including children and adults) may experience and perceive a range health and well-being impacts. Further high quality evidence is needed to facilitate subgroup analysis of health benefits and the extent of well-being benefits.

School gardens are complex interventions, yet few studies articulated a logic model to show how it was believed that school gardens might have an impact on health and wellbeing. More appropriate study design, and more consistency in the way food intake is measured, is required.
