# Erratum to: A systematic review of the health and well-being impacts of school gardening: synthesis of quantitative and qualitative evidence

**DOI:** 10.1186/s12889-016-3736-z

**Published:** 2016-10-05

**Authors:** Heather Ohly, Sarah Gentry, Rachel Wigglesworth, Alison Bethel, Rebecca Lovell, Ruth Garside

**Affiliations:** 1 European Centre for Environment and Human Health, University of Exeter Medical School, Truro, Cornwall UK; 2 Norfolk and Norwich University Hospitals NHS Foundation Trust, Norwich, Norfolk UK; 3 NIHR CLAHRC South West Peninsula (PenCLAHRC), University of Exeter Medical School, Exeter, Devon UK

## Erratum

Following the publication of this article [1] it was brought to our attention that reference 12 was incomplete.

Reference 12 currently reads: Chiumento A. A haven of greenspace. 2012.

The completed reference should read: Chiumento A. A haven of greenspace. YoungMinds Magazine 2012;118:32–34.
